# The Cross-Links of Endoplasmic Reticulum Stress, Autophagy, and Neurodegeneration in Parkinson’s Disease

**DOI:** 10.3389/fnagi.2021.691881

**Published:** 2021-06-03

**Authors:** Haigang Ren, Wanqing Zhai, Xiaojun Lu, Guanghui Wang

**Affiliations:** ^1^Department of Neurology, Center of Translational Medicine, Taicang Affiliated Hospital of Soochow University, The First People’s Hospital of Taicang, Suzhou, China; ^2^Jiangsu Key Laboratory of Translational Research and Therapy for Neuropsychiatric Disorders, Department of Pharmacology, College of Pharmaceutical Sciences, Soochow University, Suzhou, China

**Keywords:** Parkinson’s disease, ER stress, UPR, autophagy, α-synuclein, cross-link

## Abstract

Parkinson’s disease (PD) is the most common neurodegenerative movement disorder, and it is characterized by the selective loss of dopaminergic (DA) neurons in the substantia nigra pars compacta (SNpc), as well as the presence of intracellular inclusions with α-synuclein as the main component in surviving DA neurons. Emerging evidence suggests that the imbalance of proteostasis is a key pathogenic factor for PD. Endoplasmic reticulum (ER) stress-induced unfolded protein response (UPR) and autophagy, two major pathways for maintaining proteostasis, play important roles in PD pathology and are considered as attractive therapeutic targets for PD treatment. However, although ER stress/UPR and autophagy appear to be independent cellular processes, they are closely related to each other. In this review, we focused on the roles and molecular cross-links between ER stress/UPR and autophagy in PD pathology. We systematically reviewed and summarized the most recent advances in regulation of ER stress/UPR and autophagy, and their cross-linking mechanisms. We also reviewed and discussed the mechanisms of the coexisting ER stress/UPR activation and dysregulated autophagy in the lesion regions of PD patients, and the underlying roles and molecular crosslinks between ER stress/UPR activation and the dysregulated autophagy in DA neurodegeneration induced by PD-associated genetic factors and PD-related neurotoxins. Finally, we indicate that the combined regulation of ER stress/UPR and autophagy would be a more effective treatment for PD rather than regulating one of these conditions alone.

## Introduction

Parkinson’s disease (PD), the most common neurodegenerative movement disorder, affects approximately 1% of the population over 60 years old, and its incidence dramatically increases to approximately 5% in the population greater than 85 years of age (Li and Le, 2020; Wang et al., 2020b). PD is a chronic, irreversible, and complex neurodegenerative disease with several typical motor impairments including resting tremor, bradykinesia, rigidity, and postural imbalance. These are caused by the selective and progressive loss of dopaminergic (DA) neurons in the substantia nigra pars compacta (SNpc) and the deficiency of dopamine release in the striatum (Poewe et al., 2017). In addition to DA neuron loss, the presence of intracellular inclusions called Lewy bodies (LBs) with an accumulation of protein aggregates including α-synuclein (α-SYN), and abnormal dystrophic neurites termed Lewy neurites (LNs) in surviving DA neurons, are also the major hallmarks of PD pathology (Kalia and Lang, 2015). Recently, great achievements have been made in understanding PD pathogenesis, which may contribute to developing more optimal strategies for PD treatment.

The mechanisms of DA neuron damage in PD include a variety of cellular processes such as α-SYN aggregates, mitochondrial damage, oxidative stress, calcium homeostasis dysfunction, axonal transport disruption, and neuroinflammation injury, and also involve aging, and environmental and genetic factors (Shulman et al., 2011). Emerging evidence suggests that these disadvantages can perturb the balance of cellular homeostasis (proteostasis) in PD (Lehtonen et al., 2019). Endoplasmic reticulum (ER) stress-induced unfolded protein response (UPR) and autophagy, two major pathways that respond to an imbalance in cellular homeostasis, play particularly important roles in the pathology of neurodegenerative diseases including PD (Karabiyik et al., 2017; Costa et al., 2020).

Endoplasmic reticulum stress, UPR, and autophagy have become important targets for alleviating the damage of DA neurons, and they provide attractive clues for the treatment of PD (Moors et al., 2017; Martinez et al., 2019). However, UPR and autophagy are both related and independent cellular processes. Both the effects of their cross-talk and their unique mechanisms in PD must be considered in order to find an appropriate therapeutic target for PD treatment or to provide the basis for combination medication. In this review, we summarize the most recent advances in ER stress, UPR, autophagy, and their interactions in PD pathogenesis, and how to alleviate the toxic effects of cellular homeostasis imbalance in PD by targeting ER stress, UPR, and autophagy.

## ER Stress and UPR

The ER is essential for cellular homeostasis. The ER organelles have a variety of important functions that are necessary for protein synthesis, protein quality control, Ca^2+^ homeostasis, and lipid and carbohydrate metabolism (Wang and Kaufman, 2016; Ghemrawi and Khair, 2020). Under physiological states, the ER uses the resident chaperone molecules to ensure the proper folding of newly synthesized proteins, and also can identify misfolded proteins through quality control mechanisms and employ proteasomes to implement ER-associated degradation (ERAD) (Walter and Ron, 2011). When the protein-folding capacity of the ER is saturated under various pathological conditions, such as aberrant aggregation of misfolded proteins or mutant proteins, accumulation of exogenous viral proteins, fluctuation of ER Ca^2+^ stores, perturbation of ATP levels, presence of environmental toxins, or occurrence of metabolic dysfunctions, the cells sense the ER stress and subsequently initiate an adaptive response referred to as UPR. This in turn attempts to alleviate ER stress by enhancing the protein-folding capacity and reducing the general synthetic load to restore ER homeostasis and maintain cell survival (Xu et al., 2012; Rashid et al., 2015).

The UPR is controlled by three ER-resident sensors: inositol-requiring kinase 1α (IRE1α), protein kinase RNA-activated (PKR)-like ER kinase (PERK), and activating transcription factor 6 (ATF6) (Manie et al., 2014). Both IRE1α and PERK are type I transmembrane Ser/Thr protein kinases, and possess similar structures with an NH_2_-terminal ER luminal domain and a cytosolic kinase domain (Pandey et al., 2019). In addition, IRE1α has an extra RNase domain with endonuclease activity (Abdullah and Ravanan, 2018). ATF6 is a type II transmembrane protein with a COOH-terminal ER luminal domain and an NH_2_-terminal cytosolic domain with bZIP transcription factor activity (Pandey et al., 2019).

**FIGURE 1:**
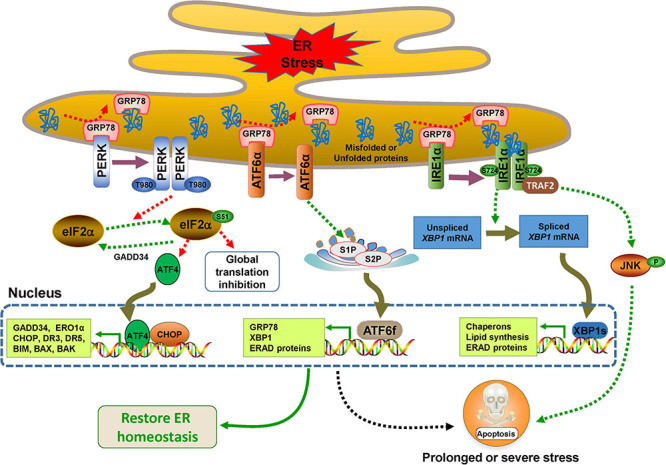
Endoplasmic reticulum (ER) stress and UPR signaling pathways. The UPR is controlled by three major branches, IRE1α, PERK, and ATF6, which bind to GRP78 in the ER under normal conditions. In response to ER stress, the three sensors are activated by dissociating with GPR78. PERK undergoes dimerization, autophosphorylation, and then decreases protein synthesis by phosphorylating eIF2α at the Ser51, which selectively results in the translation of transcription factor 4 (ATF4), a factor that activates transcription of its downstream UPR genes such as CCAAT/enhancer binding protein (C/EBP) homologous protein (CHOP) (Harding et al., 2000). CHOP and ATF4 can upregulate the expression of genes involved in the UPR and apoptosis such as BIM, BAX, BAK, death receptor 3 (DR3), DR5, inositol 1,4,5-trisphosphate (IP3) receptor 1 (IP3R1) and ER oxidase 1α (ERO1α) (Iurlaro and Munoz-Pinedo, 2016). When the ER stress is relieved, CHOP and ATF4 induce the expression of growth arrest and DNA damage inducible protein 34 (GADD34), which directly dephosphorylates eIF2α and restarts global mRNA transcription (Novoa et al., 2001). IRE1α also undergoes dimerization, and even oligomerization and autophosphorylation, and then, its RNase activity is activated and *XBP1* mRNA is cleaved to generate XBP1s. The transcription factor XBP1s is responsible for the expression of a subset of downstream genes involved in ERAD, lipid synthesis, protein folding, translocation, and secretion (Korennykh and Walter, 2012; Hetz and Papa, 2018). Activated IRE1α also interacts with tumor necrosis factor receptor (TNFR)-associated factor-2 (TRAF2) and promotes a cascade of phosphorylation events that ultimately activates Jun amino-terminal kinase (JNK)-mediated cell death (Urano et al., 2000). The dissociation of GRP78 drives ATF6 to translocate to the Golgi, where it is cleaved by site-1 protease (S1P) and S2P to generate ATF6f, which transcriptionally activates the expression of a variety of genes involved in ERAD and ER chaperones including GRP78 and XBP1 (Haze et al., 1999; Shen et al., 2002; Yamamoto et al., 2007; Hillary and FitzGerald, 2018).

In the absence of ER stress, all three UPR sensors remain inactive resulting from binding to the 78 kDa glucose-regulated protein 78 (GRP78), also known as binding-immunoglobulin protein (BIP), an abundant ER-resident chaperone encoded by gene *HSPA5*. When mutant, unfolded, or misfolded proteins accumulate in the ER, they release the inhibitory effect of GRP78 on PERK, IRE1α, and ATF6 activity through their high affinity for GRP78 (Oakes and Papa, 2015). In addition, the unfolded or misfolded proteins also act as active ligands for their activation (Credle et al., 2005; Gardner and Walter, 2011). All three UPR branches have outputs to attempt to alleviate ER stress for cell survival by distinct or partially overlapped mechanisms such as mitigating protein misfolding, reducing protein synthesis, or enhancing protein degradation (Figure 1). However, if the adaptive UPR fails to restore ER homeostasis, UPR signaling undergoes continuous activation to initiate pro-death signals through multiple pathways that eventually trigger intrinsic apoptosis (Hetz and Papa, 2018).

## Autophagy

Autophagy is a highly conserved self-degradation process that delivers aggregated or misfolded proteins, lipid droplets, glycogens, and damaged organelles to lysosomes for degradation. It plays a fundamental role in the homeostasis of almost all types of cells, tissues, and organs (Levine and Kroemer, 2019). Autophagy has been divided into at least three subtypes: macroautophagy, chaperone-mediated autophagy (CMA), and microautophagy, with each involving different mechanisms of substrate delivery to the lysosome. Microautophagy degrades the cytosolic contents through small invaginations in the lysosomal membrane, while CMA mediates selective cytoplasmic proteins with a consensus KFERQ sequence motif into the lysosomal lumen for degradation upon recognition by heat shock cognate 71 kDa protein (HSC70) and targeting by lysosomal associated membrane protein 2A (LAMP2A) (Tekirdag and Cuervo, 2018). Macroautophagy (hereafter referred to autophagy) is the major and most thoroughly characterized autophagic pathway. It sequesters cytoplasmic contents within a double-membrane structure followed by fusion with lysosomes for degradation. According to the type of substrates it degrades, autophagy is also classified into non-selective autophagy and selective autophagy (Shimizu, 2018). Selective autophagy mediates specific substrates or organelles such as aggregate-prone proteins (aggrephagy), mitochondria (mitophagy), peroxisomes (pexophagy), pathogens (xenophagy), and ER (ERphagy) to the autophagic machinery for degradation via adaptor molecules (Stolz et al., 2014).

**FIGURE 2:**
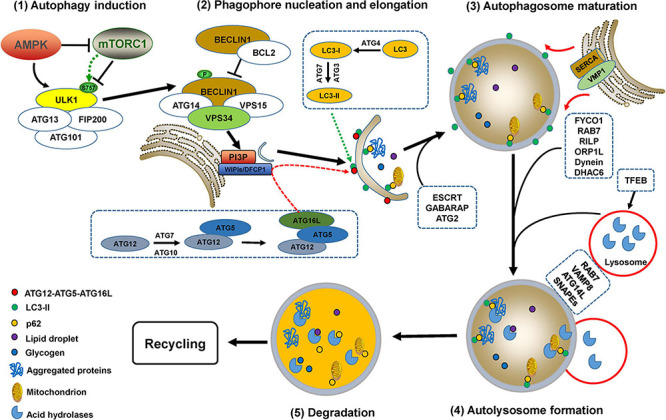
The processes of autophagy. Autophagy processes include autophagy induction, phagophore nucleation, elongation and closure, autophagosome maturation, and fusion with lysosomes to form autolysosomes, which are mediated by multiple ATG proteins and a variety of kinases or partners with complex mechanisms. Autophagy initiation requires the activation of the uncoordinated-51-like kinase (ULK) complex, which is regulated by two kinases, AMP-activated protein kinase (AMPK) and mammalian target of rapamycin (mTOR) complex 1 (mTORC1). ULK1 activation causes phosphorylation of BECLIN1 and ATG14, thus promoting the formation of the PI3KC3 complex, which enhances the activity of VPS34 to generate phosphatidylinositol 3-phosphate (PI3P) (Russell et al., 2013; Nascimbeni et al., 2017). The enrichment of PI3P recruits double FYVE domain-containing protein 1 (DFCP1), downstream ATG proteins, and WD repeat proteins interacting with phosphoinositides 1 and 2 (WIPI1/2) for phagophore nucleation (Young and Wang, 2018). Phagophore elongation is mediated by two ubiquitin-like conjugation systems, ATG12-ATG5-ATG16L and the ATG8 (light chain 3, LC3) conjugation system (Noda and Inagaki, 2015). Phagophore closure is most likely accomplished by endosomal sorting complex required for transport (ESCRT) machinery-mediated membrane abscission (Tsuboyama et al., 2016; Takahashi et al., 2018, 2019). Additionally, the γ-aminobutyric acid receptor-associated protein (GABARAP) and ATG2 family are also critical for phagophore closure (Weidberg et al., 2010; Bozic et al., 2020). The ER-localized transmembrane protein vacuole membrane protein 1 (VMP1) mediates ER-phagophore dissociation via activating ER Ca^2+^ channel sarcoplasmic Ca^2+^-ATPase (SERCA) and perturbing the local Ca^2+^ concentration (Zhao et al., 2017). Mature autophagosomes migrate to lysosomes involves the participation of proteins such as RAB7, FYVE coiled-coil domain-containing protein 1 (FYCO1), RAB-interacting lysosomal protein (RILP), Oxysterol-binding protein-related protein 1L (ORP1L), histone deacetylase 6 (HDAC6), kinesin or dynein (Nakamura and Yoshimori, 2017). RAB GTPases, soluble *N*-ethylmaleimide-sensitive factor attachment protein receptors (SNAREs), PI3K complex, and multiple tethering factors are involved in the fusion of autophagosomes with lysosomes to form autolysosomes, in which the autophagosomal contents are degraded by lysosomal acid hydrolases (Martini-Stoica et al., 2016; Zhao and Zhang, 2019). Transcription factor EB (TFEB) is a major regulator for autophagosome formation, lysosomal biogenesis, and lysosomal function (Martini-Stoica et al., 2016; Cortes and La Spada, 2019).

Under normal conditions, basal autophagy is ongoing at lower levels so that cells will optimally function after the removal of damaged organelles or unwanted substrates (Deegan et al., 2013). Autophagy is dramatically triggered by various stimuli such as nutrient deprivation, accumulation of protein aggregates, oxidative stress, hypoxia, and toxic molecule treatment (Corona Velazquez and Jackson, 2018). The complete processes of autophagy contain multiple sequential steps (autophagy induction, phagophore nucleation, elongation and closure, autophagosome maturation, and fusion with lysosomes to form autolysosomes) with complex mechanisms mediated by autophagy-related gene (ATG) proteins, their partners, and a variety of kinases (Figure 2).

## Interplay of ER Stress and Autophagy

**FIGURE 3:**
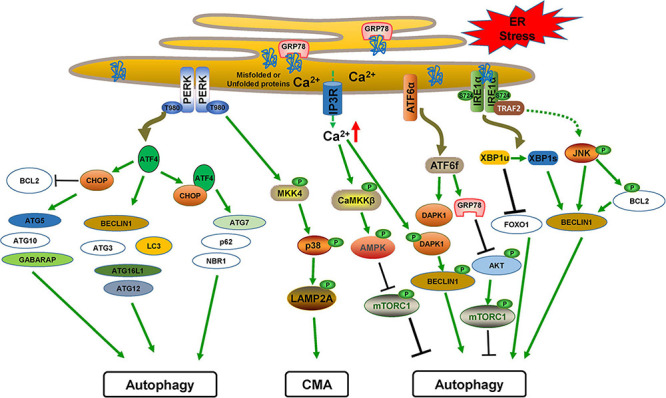
The cross-links between ER stress/UPR and autophagy. PERK-mediated ATF4 translation induces CHOP. The transcriptional factors ATF4 and CHOP induce multiple ATG genes and autophagy regulatory factors alone or together. PERK also activates the MKK4/p38 pathway for CMA activation by phosphorylating LAMP2A. ATF6f activates the expression of GRP78, which inhibits AKT/mTORC1 activation, and DAPK1, which phosphorylates BECLIN1 to induce autophagy. IRE1α activation leads to JNK signaling, which induces BECLIN1 expression and also phosphorylates BCL2 to dissociate BECLIN1 for autophagy activation. XBP1s generation by IRE1α activation also transcriptionally activates BECLIN1 expression.

Although ER stress and autophagy can act as independent drivers, they share many common features such as protecting tissues by alleviating stress and inducing cell death when alleviating failure caused by extreme or chronic stress. Since it was first described in yeast in 2006, a large number of studies have revealed that ER stress and autophagy are mechanistically related (Bernales et al., 2006; Yorimitsu et al., 2006; Rashid et al., 2015). Changing the functions of one system can affect the homeostasis of the other system, in which activation of autophagy triggered by ER stress-induced UPR is dominant. All three UPR branches can directly activate autophagy induction and autophagosome formation during ER stress through activating the expression of multiple ATG genes, inhibiting the expression of several autophagy inhibitors, or modulating several kinases including AMPK or mTORC1 (Figure 3). ER stress also regulates autophagy through mediating Ca^2+^ release. For example, ER stress stimulates Ca^2+^ release from the ER lumen into the cytosol through the IP3R channel, which activates the CaMKKβ/AMPK pathway and inhibits mTORC1 activity, thus triggering autophagy induction. In addition, the presence of Ca^2+^ in the cytosol leads to death-associated protein kinase 1 (DAPK1) activation, which phosphorylates BCL2 and BECLIN1 to induce autophagy (Verfaillie et al., 2010). However, whether ER stress affects autophagosome maturation, autolysosome formation, or lysosomal degradation is largely unknown.

### PERK and Autophagy

Activation of the PERK pathway contributes to expression of multiple ATG genes (Cai et al., 2016). Under hypoxia, PERK-mediated ATF4 and CHOP activation transcriptionally activates LC3 and ATG5 expression (Rouschop et al., 2010; Rzymski et al., 2010). *ATG12* mRNA as well as its protein levels are increased by PERK-mediated eIF2α phosphorylation (Kouroku et al., 2007). ATF4 is sufficient for transcription of several ATG genes including *ATG3*, *BECLIN1*, *LC3*, *ATG12*, and *ATG16L1*, whereas CHOP expression transcriptionally upregulates ATG5, ATG10, and GABARAP expression (B’Chir et al., 2013). ATF4/CHOP heterodimer induces the transcriptional expression of *ATG7*, as well as *p62* and neighbor of BRCA1 gene 1 (*NBR1*), which act as cargo receptors for selective autophagy of ubiquitinated targets (Lamark et al., 2009; B’Chir et al., 2013). CHOP can also promote autophagy through inhibiting the expression of BCL2, a protein that sequesters BECLIN1 in the ER and inhibits autophagosome formation (Pattingre et al., 2005; Rzymski et al., 2010).

The PERK pathway also initiates autophagy by activating AMPK and inhibiting mTORC1 activity (Avivar-Valderas et al., 2013). In addition, PERK-mediated ATF4/CHOP expression induces autophagy by inhibiting mTORC1 activity (Salazar et al., 2009; Bruning et al., 2013). Recently, The TFEB-mediated GADD34 expression may integrate mTORC1-mediated autophagy and ER stress (Gambardella et al., 2020). ER stress can also activate the CMA pathway by PERK activation, which recruits mitogen-activated protein kinase 4 (MKK4) and activates p38 to phosphorylate LAMP2A for CMA activation (Li et al., 2017).

### IRE1α and Autophagy

IRE1α activation phosphorylates c-Jun N-terminal kinase (JNK) (Ogata et al., 2006), and this activation induces autophagy by directly phosphorylating BCL2 and disrupting its interaction with BECLIN1, which induces autophagosome formation (Wei et al., 2008). Moreover, JNK activation also upregulates BECLIN1 transcription (Li et al., 2009; Ren et al., 2010). The IRE1-dependent activation of AMPK is also involved in autophagy initiation (Meares et al., 2011). The generation of XBP1s, which is dependent on IRE1α RNase activity, also induces autophagy through direct transcriptional activation of BECLIN1 expression (Margariti et al., 2013). Interestingly, the unspliced form of XBP1 (XBP1u) negatively regulates autophagy through interacting with forkhead box O1 (FOXO1) and decreasing its levels (Zhou et al., 2011; Vidal et al., 2012; Zhao et al., 2013), suggesting that IRE1α-mediated XBP1 splicing during ER stress is critical for autophagy induction.

### ATF6 and Autophagy

It has been suggested that the activation of the ATF6 branch triggers autophagy. ATF6-mediated GRP78 transcriptional expression induces autophagy initiation by inhibiting AKT activity (Yung et al., 2011). In addition, during ER stress, formed ATF6f induces the expression of DAPK1 (Kalvakolanu and Gade, 2012; Gade et al., 2014), which phosphorylates BECLIN1 so that it subsequently dissociates from its negative regulator BCL2, thus promoting autophagosome formation (Zalckvar et al., 2009).

## ER Stress, UPR, and Autophagy Cross Talk in PD Pathogenesis

Dopaminergic neurons are particularly sensitive to unfolded, misfolded, and excessively aggregated proteins. ER stress and autophagy impairment are two essential events that lead to the imbalance of proteostasis, which contributes to DA neurodegeneration. In recent years, a large number of studies have focused on the relationship between ER stress and PD pathogenesis. For example, injection of an ER stress inducer, tunicamycin, into mouse brains causes high levels of oligomeric α-SYN, DA neuron death, locomotor deficiency, and glial activation (Coppola-Segovia et al., 2017), suggesting that administration of an ER stress inducer into the SNpc could be a novel animal model of PD. However, impaired autophagy, CMA, and mitophagy are frequently observed rather than autophagy activation in PD. Interestingly, conditional deletion of the *Atg7* gene in mice recapitulates many of the pathologic features of PD, including age-related loss of DA neurons, loss of striatal DA, accumulation of α-SYN, and ubiquitinated protein aggregates (Ahmed et al., 2012). Thus far, it is generally recognized that in PD patients as well as various PD cellular and animal models, ER stress activation, UPR markers, and autophagy dysfunction undoubtedly exist in the lesion regions, and are closely related to both genetic and neurotoxic factors that induce DA neurodegeneration.

### ER Stress, UPR Activation, and Autophagy Dysfunction in Tissues of PD Patients

The UPR activation markers, including phosphorylation of PERK, eIF2α, and IRE1α, are observed in neuromelanin-containing DA neurons in the postmortem SNpc of PD patients rather than age-matched controls (Hoozemans et al., 2007; Hoozemans et al., 2012; Heman-Ackah et al., 2017). In addition, the immunoreactivity of phosphorylated PERK is co-localized with increased α-SYN immunoreactivity in DA neurons (Hoozemans et al., 2007). Importantly, UPR activation is an early event in neurodegeneration and is closely associated with the accumulation and aggregation of α-SYN (Hoozemans et al., 2012). GRP78 and CHOP, which are ER stress markers, are increased in the SNpc in PD patients (Selvaraj et al., 2012). Moreover, GRP78 is increased to a greater extent in dementia with LB (DLB) and PD with dementia (PDD) patients in the cingulate gyrus and parietal cortex (Baek et al., 2016). GRP78 is also dramatically upregulated in the brain tissues of autosomal recessive juvenile PD (AR-JP) patients caused by a loss of functional Parkin, a familial PD genetic factor (Imai et al., 2001). This is due to the defective function of Parkin’s E3 ligase activity, which results in a lack of degradation and subsequent accumulation of its substrate, the PAEL receptor.

The protein disulfide isomerase (PDI) family participates in disulfide bond formation, reduction, isomerization, and accurate folding of nascent proteins, and they also are increased to constitute an adaptive response to ER stress (Turano et al., 2002). It was found that a PDI member, pancreatic PDI (PDIp), accumulates in PD patient tissues (Conn et al., 2004). Homocysteine-induced ER protein (HERP) is a stress response protein that functions in ER folding and ERAD-mediated degradation, and ER load reduction is also increased in the SN of PD patients (Slodzinski et al., 2009). However, all PD patients do not experience an increase in the UPR in tissues. Baek et al. (2019) found that *GRP78* mRNA levels are upregulated, but they also showed that the protein levels of GRP78 are decreased in several brain regions, including the cingulate gyrus in PD patients. Recently, similar findings suggested that both GRP78 and ATF4 protein levels are decreased in the SNpc in PD patients (Esteves and Cardoso, 2020). These inconsistent results may be related to the different pathological degrees of PD patients. In the early stage of PD, UPR activation is an adaptive response to protect DA neurons from damage. However, in the late stage, excessive stress-induced neuron damage or severe DA neuron loss leads to inhibition of expression of these ER stress markers. Finally, ER stress and UPR activation are undoubted pathological processes in PD.

It was first discovered that autophagic degeneration of DA neurons occurred in the SN regions of PD patients in early studies (Anglade et al., 1997). Later, immunopositivity for LC3-II indicated that autophagosome formation occurred in the majority of LBs and LNs, and LC3-II colocalized with α-SYN in PD patients (Alvarez-Erviti et al., 2010; Dehay et al., 2010; Tanji et al., 2011), while the amount of lysosomes, and levels and activities of glucocerebrosidase (GCase) or protease cathepsin D (CTSD) were decreased within DA neurons in PD tissues (Dehay et al., 2010; Moors et al., 2019). Moreover, enlarged mitochondria have been observed within autophagosomes using confocal laser scanning microscopy in PD brains, suggesting PD-associated abnormal mitophagy (Zhu et al., 2003). Fiesel and Springer (2015); Fiesel et al. (2015) and Hou et al. (2018) confirmed that mitophagy indicated by ubiquitin Ser65 phosphorylation is specifically increased in PD patients and correlates with levels of LBs. Levels of HSC70 and LAMP2A were also dramatically decreased, which indicated that the CMA activity is significantly reduced in the SN tissues of PD patients (Alvarez-Erviti et al., 2010; Murphy et al., 2015).

The selective loss of LAMP2A protein and decreased levels of HSC70 were directly correlated with the increase in α-SYN levels and the accumulation of cytosolic CMA substrate proteins in PD samples (Murphy et al., 2015). Interestingly, crowded organelles and lipid membranes, including dystrophic lysosomes, mitochondria, and autophagosome-like structures, were observed in a recent PD postmortem study (Shahmoradian et al., 2019). Together, impairments in autophagy and CMA have been found in lesion regions in PD patients compared with matched controls using postmortem tissues. The increased autophagosomes and failed lysosomal clearance are common hallmarks in SN tissues of PD patients. The activated ER stress and UPR pathway may affect increases in autophagosome formation through the mechanisms described above. However, whether and how ER stress/UPR affects autophagosome fusion with lysosomes or lysosomal functions in PD are largely unclear.

### Association of PD Genetic Factors With ER Stress, UPR, and Autophagy

Several PD-related genetic factors including gain of function of α-SYN and LRRK2, and loss of function of Parkin, PINK1, and DJ-1, affect ER stress/UPR and autophagy, and discussing the roles and mechanisms of these genetic factors in these two biological processes can greatly promote the understanding of PD pathology.

#### α-SYN in ER Stress, UPR, and Autophagy

**FIGURE 4:**
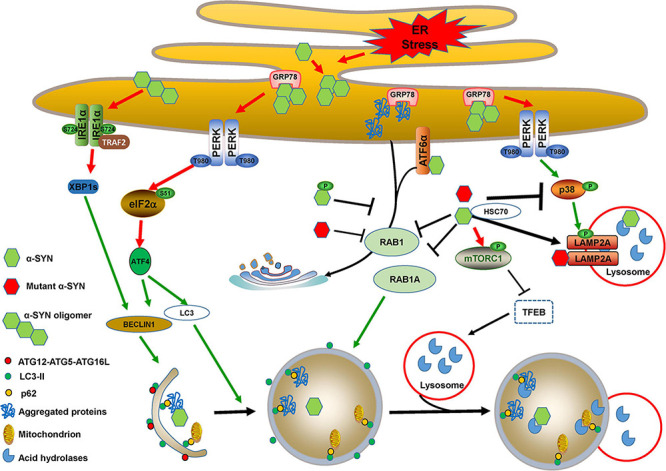
Cross-links between ER stress and autophagy in α-SYN-mediated pathology. Accumulated α-SYN binds to GRP78 and activates ER stress. α-SYN also induces ER stress by binding to ATF6 and inhibiting its translocation to Golgi bodies. Additionally, wild-type, mutant, or phosphorylated α-SYN activates ER stress by inhibiting ER-Golgi trafficking, which leads to the accumulation of aggregated proteins. PERK/ATF4 activation that occurred due to ER stress induces BECLIN1 and LC3 expression. Oligomeric α-SYN directly activates the IRE1α/XBP1s branch and thus induces BECLIN1 expression. The expression of BECLIN1 and LC3 triggers autophagy induction. However, wild-type and mutant α-SYN inhibit autophagosome maturation by repressing RAB1A function. They also activate mTORC1 and sequester TFEB in the cytoplasm to block autophagic flux by impairing lysosomal biogenesis and function. PERK activation-mediated MKK4/p38/LAMP2A phosphorylation may promote CMA for wild-type α-SYN degradation, whereas wild-type and mutant α-SYN directly inhibit p38 activation to block CMA, and mutant α-SYN can also inhibit CMA uptake by interacting with LAMP2A.

α-SYN is encoded by the *SNCA* gene, the mutations of which such as A53T, or duplication or triplication, have been linked to autosomal-dominant forms of PD. Aggregated α-SYN, especially the accumulation in the brain of its soluble oligomers, is one of most important causative factors for both hereditary and sporadic PD. α-SYN is a major component deposited in LBs and LNs, in which it harbors extensive phosphorylation at Ser129, which mediates its aggregation and toxicity (Wang et al., 2020a). α-SYN can be degraded via multiple clearance machineries, including autophagy, CMA, and the ubiquitin proteasome system (Hou et al., 2020). Although wild-type, mutant, phosphorylated, and oligomeric α-SYN activate ER stress and promote autophagy induction, they block autophagic flux by impairing autophagosome maturation, fusion with lysosomes, and lysosomal biogenesis or functions (Figure 4).

α-SYN overexpression and its aggregated neurotoxic forms activate all three UPR branches and trigger chronic ER stress-induced apoptosis. It was discovered that overexpression of both wild-type and A53T mutant α-SYN affects RAB1, which is involved in trafficking substrates from the ER to the Golgi bodies, thus inducing UPR activation by blocking ER-Golgi trafficking. RAB1 overexpression reduces stress and protects against DA neurodegeneration in PD animal models (Cooper et al., 2006), and the Ser129 phosphorylation of α-SYN may mediate the ER-Golgi traffic disruption and trigger PERK/eIF2α branch activation in *in vitro* PD models (Sugeno et al., 2008; Jiang et al., 2010). A30P α-SYN disrupts the Golgi morphology and facilitates the susceptibility to ER stress. A53T α-SYN upregulates GRP78 levels and eIF2α phosphorylation, and results in mitochondrial cell death in neurons, as well as in astrocytes (Smith et al., 2005; Liu et al., 2018; Paiva et al., 2018).

Accumulation of α-SYN within the ER activates the PERK pathway by directly interacting with GRP78 *in vitro* and *in vivo* (Bellucci et al., 2011). α-SYN oligomers rather than monomers also activate the IRE1α-XBP1 pathway (Castillo-Carranza et al., 2012). In addition, α-SYN reduces ATF6 processing and leads to ERAD impairment by directly binding to ATF6 or indirectly restricting its incorporation into coat protein complex II (COPII) vesicles (Credle et al., 2015). ER stress also leads to the accumulation of α-SYN oligomers (Jiang et al., 2010), suggesting that ER stress plays an important role in α-SYN neurotoxicity. It is interesting to hypothesize whether α-SYN toxicity is a cause or a consequence of ER stress and UPR dysfunction. Additional evidence indicates that α-SYN accumulation within the ER is required for UPR activation, and toxic α-SYN oligomer formation precedes ER stress and UPR activation (Colla et al., 2012a, b). Nevertheless, collaboration of α-SYN accumulation-induced ER stress and ER stress-enhanced α-SYN neurotoxicity is vital for PD pathogenesis.

Both wild-type and A53T mutant α-SYN can promote autophagy induction by upregulating BECLIN1 and LC3 expression (Yu et al., 2009; Decressac et al., 2013). The upregulation of BECLIN1 and LC3 expression may be involved in α-SYN-mediated ER stress and activation of the PERK/eIF2α/ATF4 pathway. However, wild-type α-SYN overexpression impairs autophagic flux via RAB1A inhibition, leads to ATG9 mislocalization, and inhibits the formation of autophagosomes (Winslow et al., 2010). RAB1 overexpression protects DA neurodegeneration in various PD animal models (Cooper et al., 2006; Gitler et al., 2008; Coune et al., 2012). Additionally, α-SYN binds to high mobility group box 1 (HMGB1) and strengthens BECLIN-BCL2 binding by blocking HMGB1-BECLIN1 interaction (Song et al., 2014). Both wild-type and A53T α-SYN can induce mTOR activity and impair autophagy (Jiang et al., 2013; Gao et al., 2015). In an AAV-mediated α-SYN overexpression mouse model, α-SYN impairs autophagic efflux by sequestering TFEB in the cytoplasm, accompanied by p62 and LC3-II accumulation (Decressac et al., 2013). Similarly, PC12 cells harboring A53T α-SYN display accumulated autophagic-vesicular structures and impaired lysosomal hydrolysis (Stefanis et al., 2001). Primary neurons seeded with α-SYN fibrils exist in phosphorylated α-SYN species with high neurotoxicity, named “pα-SYN^∗^.” These result from incomplete autophagic degradation of α-SYN, which colocalizes with GRP78 at mitochondria-associated ER membranes (Grassi et al., 2018), and this may play a role in the cross-linking between ER stress, mitochondrial fission, and mitophagy.

Interestingly, wild-type α-SYN contains a KFERQ sequence and is greatly degraded through the CMA pathway, while pathogenic α-SYN mutants act as CMA uptake inhibitors through interacting with LAMP2A on the lysosomal membrane (Cuervo et al., 2004). AAV-mediated overexpression of A53T α-SYN in neurons or A53T α-SYN in transgenic mice results in a reduction of CMA-mediated proteolysis of other substrates (Xilouri et al., 2009; Malkus and Ischiropoulos, 2012). Interestingly, modification of wild-type α-SYN by DA also impairs CMA proteolysis, similar to mutant α-SYN (Martinez-Vicente et al., 2008), which enables us to understand the selective vulnerability of the SNpc in PD. Although PERK activation has an active effect on LAMP2A through activating MKK4/p38 (Li et al., 2017), PERK activation induced by α-SYN is insufficient for activating LAMP2A activity, as both wild-type and pathogenic α-SYN have been reported that can directly inhibit p38 activation (Iwata et al., 2001). Whether wild-type and pathogenic α-SYN have an effect on phosphorylation and the activity of LAMP2A is not clear.

#### Parkin and PINK1 in ER Stress, UPR, and Autophagy

Mutations in Parkin (Kitada et al., 1998) and PTEN inducible kinase 1 (PINK1) (Valente et al., 2004a) have been identified as the most common causes of autosomal-recessive early-onset PD. *Parkin* mutations account for nearly 50% of young PD patients, and *PINK1* mutations account for 1–9% (Lucking et al., 2000; Puschmann, 2013). Parkin is an E3 ligase and functions in the ERAD of misfolded ER proteins (Shimura et al., 2000; Senft and Ronai, 2015). Parkin is upregulated by ATF4 under either ER stress or mitochondrial stress and inhibits stress-induced mitochondrial dysfunction and cell death via its E3 ligase activity (Imai et al., 2000; Bouman et al., 2011). Conversely, an accumulation of PAEL receptor, a substrate of Parkin, induces ER stress and cell death (Imai et al., 2001). Furthermore, the ER stress inhibitor salubrinal prevents rotenone-induced ER stress and cell death through the ATF4-Parkin pathway (Wu et al., 2014).

Parkin also regulates ER stress and UPR via transcription factor p53-dependent XBP1 transcription regulation (Duplan et al., 2013). It has been reported that Parkin participates in ER stress regulation in DA neurons and also in astrocytes (Ledesma et al., 2002; Singh et al., 2018). Moreover, an activation of the PERK branch of the UPR was observed that was induced by defective mitochondria, and PERK inhibition is neuroprotective in *parkin* mutant flies (Celardo et al., 2016), as well as in flies harboring mutants of *pink1*, a gene in which mutations in humans are associated with both genetic and sporadic PD (Valente et al., 2004a, b). It has been shown that PINK1 inhibits ER stress-induced damage to mouse primary cortical neurons (Li and Hu, 2015), and downregulation of ER stress response genes has been detected in aged *Pink1* knockout mice (Torres-Odio et al., 2017).

Parkin and PINK1 are two important regulators that control mitophagy and mitochondrial homeostasis (Swerdlow and Wilkins, 2020). Mitophagy, the process of removing damaged mitochondria, is compromised in PD pathogenesis, and its dysfunction is closely associated with DA neurodegeneration (Liu et al., 2019). The ATF4/CHOP heterodimer transcriptionally activates expression of p62 and NBR1 (B’Chir et al., 2013), which are two cargo adaptors involved in Parkin/PINK1-mediated mitophagy, and may be underlying the role of ER stress in mitophagy (Nguyen et al., 2016). It has been reported that the ER stress induced by tunicamycin (TM) and thapsigargin (TG) prevents Parkin loss and promotes its recruitment to the mitochondria, and also activates mitophagy during reperfusion after ischemia (Zhang et al., 2014). However, the crosslink and mechanisms between ER stress and mitophagy in PD pathogenesis are mainly unknown.

#### LRRK2 in ER Stress, UPR, and Autophagy

Mutations in the leucine-rich repeat kinase 2 (*LRKK2*) gene are the most common cause of autosomal-dominant forms of PD, as well as more than 3% of sporadic PD cases (Costa et al., 2020; Tolosa et al., 2020). LRRK2 possesses kinase function for catalyzing substrates and GTPase function for GTP-GDP hydrolysis. LRRK2 is co-localized with ER markers in DA neurons (Vitte et al., 2010), and LRKK2 depletion results in GRP78 downregulation in response to 6-hydroxydopamine (6-OHDA)-induced ER stress (Yuan et al., 2011). LRRK2 phosphorylates leucyl-tRNA synthetase (LRS), and this increases the number of misfolded proteins, causes ER stress, and induces autophagy initiation (Ho et al., 2018). LRRK2-G2019S mutation exacerbates these processes. It has recently been reported that LRRK2 regulates ER-mitochondria tethering through the PERK-mediated activation of E3 ligases, and LRKK2 mutation enhances the sensitivity to ER stress and decreases mitochondrial biogenesis (Toyofuku et al., 2020). In addition, mutant LRKK2 binds to SERCA to repress its activity, leading to ER Ca^2+^ depletion and triggering ER stress, which ultimately results in mitochondrial Ca^2+^ overload and mitochondrial dysfunction in astrocytes (Lee et al., 2019).

It has been reported that LRRK2 functions in endosomal- and vesicle-trafficking pathways, plays roles in cytoskeleton dynamics and neurite outgrowth, and regulates multiple steps of the autophagy-lysosome pathway (Madureira et al., 2020). Although wild-type and mutant LRRK2 promote autophagy by ER stress induced by the phosphorylation of LRS, they impair the autophagic degradation in an LRS-independent manner (Ho et al., 2018). LRKK2-G2019S fibroblasts exhibit higher autophagic activity levels involved in activating ERK activity rather than the mTOR pathway (Bravo-San Pedro et al., 2013). An early and transient phosphorylation of ERK1/2 is involved in ER stress activation (Arai et al., 2004), indicating that LRKK2 may regulate autophagy initiation through the ERK pathway via LRS-meditated ER stress. LRRK2 and LRRK2-G2019S also regulate p62 phosphorylation, influence its affinity to ubiquitinated cargo (Park et al., 2016; Kalogeropulou et al., 2018), and decrease autophagic protein degradation. Additionally, LRKK2 and LRKK2-G2019S inhibit autophagosome formation and autophagosome-lysosome fusion in various LRRK2-related PD models may through regulating phosphorylation of a number of RAB proteins (Madureira et al., 2020). LRKK2 pathogenic mutants also impair lysosomal function, which is detected by abnormal lysosomal morphology, abnormal cellular lysosomal localization, increased lysosomal pH, or inhibition of lysosomal enzymes (Madureira et al., 2020).

Like α-SYN, LRKK2 can also be degraded by CMA, and unlike α-SYN mutants, which increase the binding affinity of HSC70 and LAMP2A, LRRK2 mutants block the formation of the CMA translocation complex by inducing LAMP2A and HSC70 accumulation at the lysosomal membrane (Orenstein et al., 2013; Ho et al., 2020). Although it has been reported that LRRK2-G2019S binds to MKK4/7 (Zhu et al., 2013), it is unclear whether LRRK2-G2019S participates in the PERK/MKK4/p38 pathway in CMA. Together, the promotion of autophagy initiation by LRRK2 and its pathogenic mutants is partly due to ER stress and UPR activation. However, they most likely inhibit autophagic flux, as well as CMA and lysosomal functions in an ER stress-independent manner.

#### DJ-1 in ER Stress, UPR, and Autophagy

DJ-1, a protein encoded by the *PARK7* gene, assumes multiple functions including antioxidative stress and chaperone properties (Mencke et al., 2021). Mutations or deletions of DJ-1 are associated with autosomal-recessive early-onset forms of PD (Bonifati et al., 2003). DJ-1 regulates ER stress and UPR by binding to and stabilizing ATF4 mRNA under both basal and stress conditions (Yang et al., 2019). DJ-1 can also protect against ER stress-induced cell death in Neuro2a cells (Yokota et al., 2003). Moreover, it has been shown that oxidized DJ-1 can interact with N-terminal arginylated GPR78, and thus facilitate the self-polymerization of p62 and the targeting of p62-cargo complexes to phagophores under oxidative stress (Lee et al., 2018). Interestingly, DJ-1 expression is regulated under ER stress such that XBP1 directly binds to its promoter and stimulates its expression (Duplan et al., 2013).

Overexpression of DJ-1 in DA neurons and in the SN of rat brains promotes ERK-dependent autophagy. Although there are no obvious effects of DJ-1 deficiency on autophagy in SH-SY5Y cells (Gonzalez-Polo et al., 2009; Ren et al., 2010), the loss of DJ-1 perturbs paraquat-induced autophagic initiation in SH-SY5Y cells by enhancing the mTOR activity (Gonzalez-Polo et al., 2009). In addition, DJ-1 deficiency in microglia impairs autophagy-mediated p62 degradation and reduces microglial-mediated α-SYN phagocytosis (Nash et al., 2017). DJ-1 protects against DA neurodegeneration through enhancing CMA in PD animal models, SH-SY5Y cells, and astrocytes (Xu et al., 2017; De Miranda et al., 2018). Together, the upregulation of DJ-1 expression in response to ER stress may enhance the CMA or autophagic degradation of aggregated proteins, which bridges the close link between ER stress and autophagy in PD pathogenesis.

### Association of PD Neurotoxins With ER Stress and UPR

The well-known parkinsonian inducers including 1-methyl-4-phenyl-1,2,3,6-tetrahydropyridine (MPTP)/1-methyl-4-phenylpyridinium (MPP^+^), 6-OHDA, and rotenone, all have profound effects on the regulation of ER stress/UPR and autophagy. Discussing the cross-link between ER stress/UPR and autophagy in these parkinsonian inducers may promote our understanding of PD pathology.

#### MPTP/MPP^+^ in ER Stress, UPR, and Autophagy

MPTP is the best-known chemical for inducing a PD model *in vivo*. Intraperitoneally injecting mice with MPTP reproduces PD pathology, including the selective loss of DA neurons in the SN and accumulation of protein aggregates, and eventually leads to the onset of PD-like clinical symptoms (Bove et al., 2005). MPTP can efficiently cross the blood-brain barrier and is metabolized to the destructive MPP^+^ by glial monoamine oxidase B (MAO-B). MPP^+^ is transported into DA neurons via dopamine transporter (DAT) and induces DA neuron death by inhibiting mitochondrial functions and increasing mitochondrial superoxide.

MPP^+^ triggers a significant increase in the expression of UPR genes including ATF4 and CHOP, which are involved in the activation of IRE1α and PERK in various cellular models (Ryu et al., 2002; Holtz and O’Malley, 2003). MPP^+^ has been shown to activate cyclin-dependent-like kinase 5 (CDK5)-mediated XBP1s phosphorylation, which favors its nuclear translocation and promotes its transcriptional activity in rat primary cultured neurons (Jiao et al., 2017). The intracerebral injection of MPP^+^ into the SNpc of rabbit brains induces ER stress involving the activation of the ATF6 and NF-κB signaling pathways (Ghribi et al., 2003). MPTP also promotes the phosphorylation of p38 and enhances the interaction between phosphorylated p38 and ATF6, leading to an increase in ATF6 transcriptional activity (Egawa et al., 2011). MPP^+^ also activates the expression of UPR markers such as PDIp, which accumulates in PD patient tissues (Conn et al., 2004). MPTP/MPP^+^ activate ER stress and expression of the UPR markers GRP78 and CHOP by disturbing ER Ca^2+^ levels, which is accompanied by a decrease in AKT/mTOR activity (Selvaraj et al., 2012), and is closely associated with autophagy activation.

The regulatory effects of MPTP/MPP^+^ on autophagy are not consistent among different studies. MPTP/MPP^+^ treatment increases autophagosome formation, increases p62 levels, and decreases lysosomal activity (Dehay et al., 2010; Lim et al., 2011; Lim et al., 2014; Jovanovic-Tucovic et al., 2019), indicating that autophagy initiation is activated but autophagic flux is blocked by MPP^+^ treatment. The activation of autophagy initiation by MPP^+^ treatment probably occurs through activation of AMPK (Jovanovic-Tucovic et al., 2019). Further study indicates that mild MPP^+^ exposure (10 or 200 μM for 48 h) predominantly inhibits autophagic degradation by reducing lysosomal hydrolase cathepsin D activity, whereas acute MPP^+^ treatment (2.5 and 5 mM for 24 h) inhibits both autophagic degradation and basal autophagy by decreasing lysosomal density (Miyara et al., 2016). MPP^+^ treatment increases autophagy activation and promotes mitochondrial degradation, which involves the activation of ERK signaling rather than the canonical pathway (Zhu et al., 2007, 2012; Dagda et al., 2008).

We speculate that MPP^+^ treatment-induced mitochondrial degradation may not be due to the activation of autophagic flux, but it dramatically disrupts mitochondrial functions and impairs mitochondrial biogenesis (Zhu et al., 2012). MPP^+^ treatment-induced ER stress and the UPR closely contribute to autophagy induction and autophagic flux blockage. All branches of the UPR are activated by MPTP/MPP^+^ treatment, as well as subsequent events such as activation of AMPK (Jovanovic-Tucovic et al., 2019), decreased mTOR activity (Selvaraj et al., 2012), and increased NF-κB signaling (Ghribi et al., 2003), which contribute to autophagy initiation (Zhu et al., 2017). MPTP/MPP^+^ treatment also disrupts lysosomal functions and leads to autophagic flux blockage (Dehay et al., 2010; Lim et al., 2011). The cross-link between autophagy initiation activation and autophagic flux blockage exaggerates the damage to DA neurons by MPTP/MPP^+^ treatment.

#### 6-OHDA in ER Stress, UPR, and Autophagy

6-OHDA, a selective catecholaminergic neurotoxin, is also widely used to induce DA neuron death as a PD model by eliciting the production of mitochondrial and cytosolic reactive oxygen species (ROS) (Simola et al., 2007). Increased levels of GRP78 and CHOP expression, as well as phosphorylated PERK and eIF2α, are detected in DA cellular models that are subjected to 6-OHDA treatment (Ryu et al., 2002; Holtz and O’Malley, 2003; Yamamuro et al., 2006; Deng et al., 2012; Xie et al., 2012; Ning et al., 2019b).

Like MPP^+^, 6-OHDA treatment also elicits autophagy activation and promotes mitochondrial degradation involving the activation of ERK signaling (Dagda et al., 2008). Unlike MPP^+^, 6-OHDA induces AMPK phosphorylation, followed by mTOR dephosphorylation, and increases LC3 conversion, p62 degradation, and cytoplasmatic acidification in SH-SY5Y cells (Arsikin et al., 2012). A recent study indicated that 6-OHDA treatment induces excessive autophagy with increased AMPK activity, decreased mTOR activity, reduced p62 levels, and also prevents alterations in lysosomal functions (Chung et al., 2018). In addition, 6-OHDA treatment stimulates CMA activity by increasing LAMP2A levels (Wang et al., 2018). 6-OHDA treatment induces BECLIN1 and decreases BCL2 expression and p62 levels, which are inhibited by PERK inhibition (Ning et al., 2019a, b), and this directly cross-links the ER stress and autophagy in 6-OHDA-induced PD pathogenesis. Together, 6-OHDA-induced excessive autophagy activation and autophagic flux contribute to PD pathogenesis.

#### Rotenone in ER Stress, UPR, and Autophagy

Rotenone treatment produces most of the movement disorder symptoms and the histopathological features of PD, including LBs (Betarbet et al., 2002). Rotenone also triggers ATF4 and CHOP expression involving activation of IRE1α and PERK in cellular models (Ryu et al., 2002; Ramalingam et al., 2019). An induction of the IRE1α and PERK branch of the UPR has also been shown in rotenone rat or mouse models of PD (Tong et al., 2016a, b; Ramalingam et al., 2019). Treatment of N2a cells with rotenone triggers ER stress and the UPR involving all three branches of PERK, IRE1α, and ATF6 (Gupta et al., 2019).

Rotenone induces an increase in autophagy related proteins LC3-II and BECLIN1, as well as in autophagy substrates such as α-SYN and p62 in cultured PC12 cells (Wu et al., 2015) and in SH-SY5Y cells (Xiong et al., 2013), suggesting that rotenone exerts bidirectional effects on autophagy initiation and autophagic flux. Rotenone increases oligomeric wild-type and A53T α-SYN in transfected cells through inhibiting their autophagic degradation (Yu et al., 2009). Similar to MPP^+^, although rotenone treatment results in decreased autophagic flux, it increases mitophagy for mitochondrial degradation (Giordano et al., 2014), suggesting that the mitochondrial-specific degradation pathway used by MPP^+^ and rotenone may be independent from that of autophagy. Therefore, rotenone-induced ER stress and the UPR initiate autophagy induction but block autophagic flux by impairing lysosomal functions, which aggravates the imbalance of cellular homeostasis and damage to DA neurons.

## Future Perspectives

**FIGURE 5:**
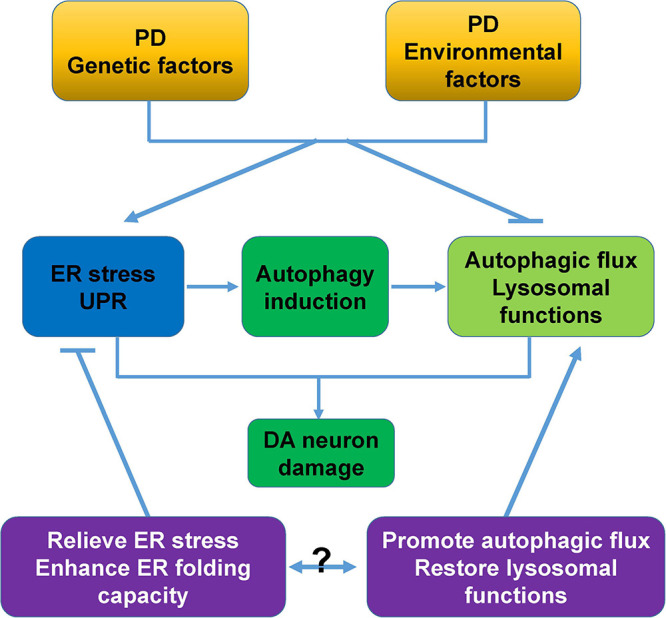
Proposed model of cross-links between ER stress, autophagy, and DA neurodegeneration. PD-associated genetic and environmental factors trigger ER stress, and ER stress activates the UPR and induces autophagy to alleviate cellular stress. However, these PD-associated factors commonly block autophagic flux and impair lysosomal functions, and these changes synergistically cause severe damage and degeneration of DA neurons. Relieving ER stress/UPR, enhancing the ER folding capacity, promoting autophagic flux, and restoring lysosomal functions are neuroprotective for PD. The combined intervention of ER stress/UPR and autophagy would be a more attractive therapeutic approach for PD.

The accumulation of unfolded, misfolded, and aggregated proteins, and the accumulation induced by cellular stress are essential mechanisms underlying the causes of PD. ER stress/UPR and autophagy, two major pathways that are used to respond to proteostasis imbalance, play especially important roles in PD pathology. In this review, we systematically examined the intrinsic molecular links between ER stress, the UPR, and autophagy, as well as the roles of these cross-links in PD pathology. ER stress, UPR activation, and dysregulated autophagy commonly coexist in patients and various cellular and animal models of PD, and are closely related to DA neurodegeneration caused by PD genetic and neurotoxic factors (Table 1). This is why targeting one of these processes would create a beneficial PD treatment (Moors et al., 2017; Martinez et al., 2019). More importantly, combining both ER stress/UPR and autophagy regulation, such as relieving ER stress/UPR or enhancing the ER folding capacity, and promoting autophagic flux or restoring lysosomal functions will be more neuroprotective for PD (Figure 5). In addition, the ER stress/UPR activation, upregulation of UPR genes, and accumulation of autophagosome markers and autophagic substrates are expected to be useful biomarkers or diagnostic parameters of PD.

**TABLE 1:** The roles and mechanisms of PD-related factors in ER stress, autophagy and their cross-links.

**PD-related factors**	**Roles and mechanisms in ER stress**	**Roles and mechanisms in autophagy**	**Cross-links**
α-SYN	α-SYN directly binds to GRP78 to activate PERK and α-SYN reduces ATF6 processing; α-SYN oligomers activate the IRE1α-XBP1 pathway; Wild-type and mutant α-SYN affect RAB1 and disrupts traffic from the ER to the Golgi; ER stress leads to the accumulation of α-SYN oligomers.	α-SYN promotes autophagy induction by upregulating BECLIN1 and LC3 expression; α-SYN impairs autophagic flux via RAB1A inhibition, TFEB sequestration and lysosomal inhibition; α-SYN is degraded via autophagy and CMA; Oxidized α-SYN and A53T α-SYN reduce CMA-mediated proteolysis through binding to LAMP2A or inhibit p38 activation.	α-SYN-mediated BECLIN1 and LC3 expression via ER stress activation may be involved in its role in autophagy induction; The inhibition of RAB pathway by α-SYN is both involved in ER stress activation and autophagic flux impairment.
Parkin/PINK1	Parkin is upregulated by ER stress; Parkin inhibits PERK-mediated ER stress through its E3 ligase activity; Parkin also regulates ER stress via p53-XBP1 pathway; PINK1 inhibits ER stress via PERK branch.	Parkin and PINK1 control mitochondrial homeostasis by enhancing mitophagy.	PERK/ATF4/CHOP induced Parkin, p62 and NBR expression may be involved in Parkin/PINK1-mediated mitophagy.
LRRK2	LRRK2 is partly localized in ER; LRRK2 and its pathogenic mutant G2019S phosphorylate LRS, and cause ER stress; Mutant LRKK2 binds to SERCA and leads to ER Ca^2+^ depletion to trigger ER stress;	LRRK2 and LRKK2-G2019S induce autophagy initiation by phosphorylating LRS; LRRK2-G2019S promotes autophagy by activating ERK; LRRK2 and LRRK2-G2019S decrease autophagic degradation by regulating p62 phosphorylation, as well as inhibit autophagosome and autolysosome formation via phosphorylating a number of RAB proteins.	LRKK2 and it mutants trigger autophagy initiation by activating ERK via LRS-meditated accumulation of misfolded proteins and ER stress. LRKK2 and it mutants activate ER stress and impair autophagic flux may also through regulating RABs functions.
DJ-1	DJ-1 regulates ER stress/UPR by binding to and stabilizing ATF4 mRNA; Oxidized DJ-1 interact with arginylated GPR78; DJ-1 is upregulated under ER stress through XBP1 branch.	DJ-1 promotes ERK-dependent autophagy; Loss of DJ-1 perturbs paraquat-induced autophagic initiation by enhancing the mTOR activity; DJ-1 deficiency in microglia impairs autophagy-mediated p62 degradation and reduces microglial-mediated α-SYN phagocytosis. DJ-1 enhances CMA activity	Oxidized DJ-1 interact with arginylated GPR78 and facilitate p62-cargo complexes to phagophore; ER stress induced DJ-1 upregulation enhances the CMA or autophagic degradation.
MPTP/MPP^+^	MPTP/MPP^+^ activates IRE1α, PERK, and ATF6 branches through enhancing CDK5 and p38 activity, as well as disturbing ER Ca^2+^ levels.	MPTP/MPP^+^ treatment increases autophagy initiation but blocks autophagic flux, probably through activating AMPK and ERK activity, and reducing mTOR activity and lysosomal hydrolase activity.	MPTP/MPP^+^ treatment-induced ER stress and the UPR activation contribute to autophagy induction.
6-OHDA	6-OHDA activates ER stress/UPR by phosphorylating PERK and eIF2α.	6-OHDA treatment elicits autophagy activation by activating ERK and AMPK activity, as well as BECLIN1 expression; 6-OHDA promotes autophagic flux and CMA activity.	6-OHDA-activated ER stress/UPR contributes to excessive autophagy initiation and autophagic flux.
Rotenone	Rotenone triggers ER stress involving activation of all three branches of PERK, IRE1α, and ATF6.	Rotenone treatment increases autophagy induction but inhibits autophagic flux by impairing lysosomal functions; rotenone increases mitophagy.	Rotenone-mediated ER stress/UPR stimulates autophagy induction.

For example, administration of GSK2606414, a PERK inhibitor, results in effective neuroprotection and prevents loss of SNpc DA neurons in mice that were treated with PD neurotoxin 6-OHDA (Mercado et al., 2018). Gene therapy that restores the folding capacity by administration of viral-mediated overexpression of GRP78 (Gorbatyuk et al., 2012) or UPR transcriptional factor XBP1s (Sado et al., 2009; Valdes et al., 2014) in the SNpc results in neuroprotection against neurotoxin- or genetic factors-induced damage to DA neurons. Rapamycin, an inhibitor of mTOR, initiates autophagy induction, enhances autophagic flux (Rubinsztein and Nixon, 2010), and confers significant protective effects on DA neurons in various PD models (Moors et al., 2017). Additionally, enhancing lysosomal biogenesis by TFEB overexpression or pharmacological stimulation of TFEB function by CCI-779 was shown to eliminate α-SYN oligomers and rescue midbrain DA neurons from α-SYN toxicity in rats (Decressac et al., 2013).

It is notable that PD-associated genetic or environmental factors lead to ER stress and UPR activation, which commonly initiate autophagy. However, these PD-associated factors also block autophagic flux and impair lysosomal functions. An intervention strategy for one of the two processes alone may not completely alleviate the imbalance in cellular homeostasis. However, a combined treatment strategy for both ER stress/UPR and the autophagy pathway has not yet been studied in PD. We recognize that combining ER stress/UPR and autophagy to explore the pathological mechanisms of PD and develop interventional strategies that combine ER stress/UPR and autophagy will be very meaningful in the future.

## Author Contributions

GW and XL designed the theme of the manuscript. HR and WZ wrote the manuscript. All authors approved the submitted version.

## Conflict of Interest

The authors declare that the research was conducted in the absence of any commercial or financial relationships that could be construed as a potential conflict of interest.

## References

[B1] Abdullah A. Ravanan P. (2018). The unknown face of IRE1alpha - beyond ER stress. *Eur. J. Cell Biol.* 97 359–368. 10.1016/j.ejcb.2018.05.002 29747876

[B2] Ahmed I. Liang Y. Schools S. Dawson V. L. Dawson T. M. Savitt J. M. (2012). Development and characterization of a new Parkinson’s disease model resulting from impaired autophagy. *J. Neurosci.* 32 16503–16509. 10.1523/JNEUROSCI.0209-12.2012 23152632 PMC3508432

[B3] Alvarez-Erviti L. Rodriguez-Oroz M. C. Cooper J. M. Caballero C. Ferrer I. Obeso J. A. (2010). Chaperone-mediated autophagy markers in Parkinson disease brains. *Arch. Neurol.* 67 1464–1472. 10.1001/archneurol.2010.198 20697033

[B4] Anglade P. Vyas S. Javoy-Agid F. Herrero M. T. Michel P. P. Marquez J. (1997). Apoptosis and autophagy in nigral neurons of patients with Parkinson’s disease. *Histol. Histopathol.* 12 25–31. 9046040

[B5] Arai K. Lee S. R. van Leyen K. Kurose H. Lo E. H. (2004). Involvement of ERK MAP kinase in endoplasmic reticulum stress in SH-SY5Y human neuroblastoma cells. *J. Neurochem.* 89 232–239. 10.1111/j.1471-4159.2004.02317.x 15030407

[B6] Arsikin K. Kravic-Stevovic T. Jovanovic M. Ristic B. Tovilovic G. Zogovic N. (2012). Autophagy-dependent and -independent involvement of AMP-activated protein kinase in 6-hydroxydopamine toxicity to SH-SY5Y neuroblastoma cells. *Biochim. Biophys. Acta* 1822 1826–1836. 10.1016/j.bbadis.2012.08.006 22917563

[B7] Avivar-Valderas A. Bobrovnikova-Marjon E. Alan Diehl J. Bardeesy N. Debnath J. Aguirre-Ghiso J. A. (2013). Regulation of autophagy during ECM detachment is linked to a selective inhibition of mTORC1 by PERK. *Oncogene* 32 4932–4940. 10.1038/onc.2012.512 23160380 PMC3600386

[B8] Baek J. H. Mamula D. Tingstam B. Pereira M. He Y. Svenningsson P. (2019). GRP78 level is altered in the brain, but not in plasma or cerebrospinal fluid in Parkinson’s disease patients. *Front. Neurosci.* 13:697. 10.3389/fnins.2019.00697 31333410 PMC6624451

[B9] Baek J. H. Whitfield D. Howlett D. Francis P. Bereczki E. Ballard C. (2016). Unfolded protein response is activated in Lewy body dementias. *Neuropathol. Appl. Neurobiol.* 42 352–365. 10.1111/nan.12260 26202523

[B10] B’Chir W. Maurin A. C. Carraro V. Averous J. Jousse C. Muranishi Y. (2013). The eIF2alpha/ATF4 pathway is essential for stress-induced autophagy gene expression. *Nucleic Acids Res.* 41 7683–7699. 10.1093/nar/gkt563 23804767 PMC3763548

[B11] Bellucci A. Navarria L. Zaltieri M. Falarti E. Bodei S. Sigala S. (2011). Induction of the unfolded protein response by alpha-synuclein in experimental models of Parkinson’s disease. *J. Neurochem.* 116 588–605. 10.1111/j.1471-4159.2010.07143.x 21166675

[B12] Bernales S. McDonald K. L. Walter P. (2006). Autophagy counterbalances endoplasmic reticulum expansion during the unfolded protein response. *PLoS Biol.* 4:e423. 10.1371/journal.pbio.0040423 17132049 PMC1661684

[B13] Betarbet R. Sherer T. B. Greenamyre J. T. (2002). Animal models of Parkinson’s disease. *Bioessays* 24 308–318. 10.1002/bies.10067 11948617

[B14] Bonifati V. Rizzu P. van Baren M. J. Schaap O. Breedveld G. J. Krieger E. (2003). Mutations in the DJ-1 gene associated with autosomal recessive early-onset parkinsonism. *Science* 299 256–259. 10.1126/science.1077209 12446870

[B15] Bouman L. Schlierf A. Lutz A. K. Shan J. Deinlein A. Kast J. (2011). Parkin is transcriptionally regulated by ATF4: evidence for an interconnection between mitochondrial stress and ER stress. *Cell Death Differ.* 18 769–782. 10.1038/cdd.2010.142 21113145 PMC3131924

[B16] Bove J. Prou D. Perier C. Przedborski S. (2005). Toxin-induced models of Parkinson’s disease. *NeuroRx* 2 484–494. 10.1602/neurorx.2.3.484 16389312 PMC1144492

[B17] Bozic M. van den Bekerom L. Milne B. A. Goodman N. Roberston L. Prescott A. R. (2020). A conserved ATG2-GABARAP family interaction is critical for phagophore formation. *EMBO Rep.* 21:e48412. 10.15252/embr.201948412 32009292 PMC7054675

[B18] Bravo-San Pedro J. M. Niso-Santano M. Gomez-Sanchez R. Pizarro-Estrella E. Aiastui-Pujana A. Gorostidi A. (2013). The LRRK2 G2019S mutant exacerbates basal autophagy through activation of the MEK/ERK pathway. *Cell. Mol. Life Sci.* 70 121–136. 10.1007/s00018-012-1061-y 22773119 PMC11113213

[B19] Bruning A. Rahmeh M. Friese K. (2013). Nelfinavir and bortezomib inhibit mTOR activity via ATF4-mediated sestrin-2 regulation. *Mol. Oncol.* 7 1012–1018. 10.1016/j.molonc.2013.07.010 23916134 PMC5528439

[B20] Cai Y. Arikkath J. Yang L. Guo M. L. Periyasamy P. Buch S. (2016). Interplay of endoplasmic reticulum stress and autophagy in neurodegenerative disorders. *Autophagy* 12 225–244. 10.1080/15548627.2015.1121360 26902584 PMC4835965

[B21] Castillo-Carranza D. L. Zhang Y. Guerrero-Munoz M. J. Kayed R. Rincon-Limas D. E. Fernandez-Funez P. (2012). Differential activation of the ER stress factor XBP1 by oligomeric assemblies. *Neurochem. Res.* 37 1707–1717. 10.1007/s11064-012-0780-7 22528838 PMC3387497

[B22] Celardo I. Costa A. C. Lehmann S. Jones C. Wood N. Mencacci N. E. (2016). Mitofusin-mediated ER stress triggers neurodegeneration in pink1/parkin models of Parkinson’s disease. *Cell Death Dis.* 7:e2271. 10.1038/cddis.2016.173 27336715 PMC5143399

[B23] Chung Y. Lee J. Jung S. Lee Y. Cho J. W. Oh Y. J. (2018). Dysregulated autophagy contributes to caspase-dependent neuronal apoptosis. *Cell Death Dis.* 9:1189. 10.1038/s41419-018-1229-y 30538224 PMC6289995

[B24] Colla E. Coune P. Liu Y. Pletnikova O. Troncoso J. C. Iwatsubo T. (2012a). Endoplasmic reticulum stress is important for the manifestations of alpha-synucleinopathy in vivo. *J. Neurosci.* 32 3306–3320. 10.1523/JNEUROSCI.5367-11.2012 22399753 PMC3461828

[B25] Colla E. Jensen P. H. Pletnikova O. Troncoso J. C. Glabe C. Lee M. K. (2012b). Accumulation of toxic alpha-synuclein oligomer within endoplasmic reticulum occurs in alpha-synucleinopathy in vivo. *J. Neurosci.* 32 3301–3305. 10.1523/JNEUROSCI.5368-11.2012 22399752 PMC3548448

[B26] Conn K. J. Gao W. McKee A. Lan M. S. Ullman M. D. Eisenhauer P. B. (2004). Identification of the protein disulfide isomerase family member PDIp in experimental Parkinson’s disease and Lewy body pathology. *Brain Res.* 1022 164–172. 10.1016/j.brainres.2004.07.026 15353226

[B27] Cooper A. A. Gitler A. D. Cashikar A. Haynes C. M. Hill K. J. Bhullar B. (2006). Alpha-synuclein blocks ER-Golgi traffic and Rab1 rescues neuron loss in Parkinson’s models. *Science* 313 324–328. 10.1126/science.1129462 16794039 PMC1983366

[B28] Coppola-Segovia V. Cavarsan C. Maia F. G. Ferraz A. C. Nakao L. S. Lima M. M. (2017). ER stress induced by tunicamycin triggers alpha-synuclein oligomerization, dopaminergic neurons death and locomotor impairment: a new model of Parkinson’s disease. *Mol. Neurobiol.* 54 5798–5806. 10.1007/s12035-016-0114-x 27660269

[B29] Corona Velazquez A. F. Jackson W. T. (2018). So many roads: the multifaceted regulation of autophagy induction. *Mol. Cell. Biol.* 38:e00303-18. 10.1128/MCB.00303-18 30126896 PMC6189458

[B30] Cortes C. J. La Spada A. R. (2019). TFEB dysregulation as a driver of autophagy dysfunction in neurodegenerative disease: molecular mechanisms, cellular processes, and emerging therapeutic opportunities. *Neurobiol. Dis.* 122 83–93. 10.1016/j.nbd.2018.05.012 29852219 PMC6291370

[B31] Costa C. A. D. Manaa W. E. Duplan E. Checler F. (2020). The endoplasmic reticulum stress/unfolded protein response and their contributions to Parkinson’s disease physiopathology. *Cells* 9:2495. 10.3390/cells9112495 33212954 PMC7698446

[B32] Coune P. G. Schneider B. L. Aebischer P. (2012). Parkinson’s disease: gene therapies. *Cold Spring Harb. Perspect. Med.* 2:a009431. 10.1101/cshperspect.a009431 22474617 PMC3312404

[B33] Credle J. J. Finer-Moore J. S. Papa F. R. Stroud R. M. Walter P. (2005). On the mechanism of sensing unfolded protein in the endoplasmic reticulum. *Proc. Natl. Acad. Sci. U.S.A.* 102 18773–18784. 10.1073/pnas.0509487102 16365312 PMC1316886

[B34] Credle J. J. Forcelli P. A. Delannoy M. Oaks A. W. Permaul E. Berry D. L. (2015). alpha-Synuclein-mediated inhibition of ATF6 processing into COPII vesicles disrupts UPR signaling in Parkinson’s disease. *Neurobiol. Dis.* 76 112–125. 10.1016/j.nbd.2015.02.005 25725420

[B35] Cuervo A. M. Stefanis L. Fredenburg R. Lansbury P. T. Sulzer D. (2004). Impaired degradation of mutant alpha-synuclein by chaperone-mediated autophagy. *Science* 305 1292–1295. 10.1126/science.1101738 15333840

[B36] Dagda R. K. Zhu J. Kulich S. M. Chu C. T. (2008). Mitochondrially localized ERK2 regulates mitophagy and autophagic cell stress: implications for Parkinson’s disease. *Autophagy* 4 770–782. 10.4161/auto.6458 18594198 PMC2574804

[B37] De Miranda B. R. Rocha E. M. Bai Q. El Ayadi A. Hinkle D. Burton E. A. (2018). Astrocyte-specific DJ-1 overexpression protects against rotenone-induced neurotoxicity in a rat model of Parkinson’s disease. *Neurobiol. Dis.* 115 101–114. 10.1016/j.nbd.2018.04.008 29649621 PMC5943150

[B38] Decressac M. Mattsson B. Weikop P. Lundblad M. Jakobsson J. Bjorklund A. (2013). TFEB-mediated autophagy rescues midbrain dopamine neurons from alpha-synuclein toxicity. *Proc. Natl. Acad. Sci. U.S.A.* 110 E1817–E1826. 10.1073/pnas.1305623110 23610405 PMC3651458

[B39] Deegan S. Saveljeva S. Gorman A. M. Samali A. (2013). Stress-induced self-cannibalism: on the regulation of autophagy by endoplasmic reticulum stress. *Cell. Mol. Life Sci.* 70 2425–2441. 10.1007/s00018-012-1173-4 23052213 PMC11113399

[B40] Dehay B. Bove J. Rodriguez-Muela N. Perier C. Recasens A. Boya P. (2010). Pathogenic lysosomal depletion in Parkinson’s disease. *J. Neurosci.* 30 12535–12544. 10.1523/JNEUROSCI.1920-10.2010 20844148 PMC6633458

[B41] Deng C. Tao R. Yu S. Z. Jin H. (2012). Inhibition of 6-hydroxydopamine-induced endoplasmic reticulum stress by sulforaphane through the activation of Nrf2 nuclear translocation. *Mol. Med. Rep.* 6 215–219. 10.3892/mmr.2012.894 22552270

[B42] Duplan E. Giaime E. Viotti J. Sevalle J. Corti O. Brice A. (2013). ER-stress-associated functional link between Parkin and DJ-1 via a transcriptional cascade involving the tumor suppressor p53 and the spliced X-box binding protein XBP-1. *J. Cell Sci.* 126(Pt 9) 2124–2133. 10.1242/jcs.127340 23447676 PMC6518232

[B43] Egawa N. Yamamoto K. Inoue H. Hikawa R. Nishi K. Mori K. (2011). The endoplasmic reticulum stress sensor, ATF6alpha, protects against neurotoxin-induced dopaminergic neuronal death. *J. Biol. Chem.* 286 7947–7957. 10.1074/jbc.M110.156430 21131360 PMC3048681

[B44] Esteves A. R. Cardoso S. M. (2020). Differential protein expression in diverse brain areas of Parkinson’s and Alzheimer’s disease patients. *Sci. Rep.* 10:13149. 10.1038/s41598-020-70174-z 32753661 PMC7403590

[B45] Fiesel F. C. Ando M. Hudec R. Hill A. R. Castanedes-Casey M. Caulfield T. R. (2015). (Patho-)physiological relevance of PINK1-dependent ubiquitin phosphorylation. *EMBO Rep.* 16 1114–1130. 10.15252/embr.201540514 26162776 PMC4576981

[B46] Fiesel F. C. Springer W. (2015). Disease relevance of phosphorylated ubiquitin (p-S65-Ub). *Autophagy* 11 2125–2126. 10.1080/15548627.2015.1091912 26389970 PMC4824591

[B47] Gade P. Manjegowda S. B. Nallar S. C. Maachani U. B. Cross A. S. Kalvakolanu D. V. (2014). Regulation of the death-associated protein kinase 1 expression and autophagy via ATF6 requires apoptosis signal-regulating kinase 1. *Mol. Cell. Biol.* 34 4033–4048. 10.1128/MCB.00397-14 25135476 PMC4386459

[B48] Gambardella G. Staiano L. Moretti M. N. De Cegli R. Fagnocchi L. Di Tullio G. (2020). GADD34 is a modulator of autophagy during starvation. *Sci. Adv.* 6:eabb0205. 10.1126/sciadv.abb0205 32978159 PMC7518873

[B49] Gao S. Duan C. Gao G. Wang X. Yang H. (2015). Alpha-synuclein overexpression negatively regulates insulin receptor substrate 1 by activating mTORC1/S6K1 signaling. *Int. J. Biochem. Cell Biol.* 64 25–33. 10.1016/j.biocel.2015.03.006 25813876

[B50] Gardner B. M. Walter P. (2011). Unfolded proteins are Ire1-activating ligands that directly induce the unfolded protein response. *Science* 333 1891–1894. 10.1126/science.1209126 21852455 PMC3202989

[B51] Ghemrawi R. Khair M. (2020). Endoplasmic reticulum stress and unfolded protein response in neurodegenerative diseases. *Int. J. Mol. Sci.* 21:6127. 10.3390/ijms21176127 32854418 PMC7503386

[B52] Ghribi O. Herman M. M. Pramoonjago P. Savory J. (2003). MPP+ induces the endoplasmic reticulum stress response in rabbit brain involving activation of the ATF-6 and NF-kappaB signaling pathways. *J. Neuropathol. Exp. Neurol.* 62 1144–1153. 10.1093/jnen/62.11.1144 14656072

[B53] Giordano S. Dodson M. Ravi S. Redmann M. Ouyang X. Darley Usmar V. M. (2014). Bioenergetic adaptation in response to autophagy regulators during rotenone exposure. *J. Neurochem.* 131 625–633. 10.1111/jnc.12844 25081478 PMC4454361

[B54] Gitler A. D. Bevis B. J. Shorter J. Strathearn K. E. Hamamichi S. Su L. J. (2008). The Parkinson’s disease protein alpha-synuclein disrupts cellular Rab homeostasis. *Proc. Natl. Acad. Sci. U.S.A.* 105 145–150. 10.1073/pnas.0710685105 18162536 PMC2224176

[B55] Gonzalez-Polo R. Niso-Santano M. Moran J. M. Ortiz-Ortiz M. A. Bravo-San Pedro J. M. Soler G. (2009). Silencing DJ-1 reveals its contribution in paraquat-induced autophagy. *J. Neurochem.* 109 889–898. 10.1111/j.1471-4159.2009.06020.x 19425177

[B56] Gorbatyuk M. S. Shabashvili A. Chen W. Meyers C. Sullivan L. F. Salganik M. (2012). Glucose regulated protein 78 diminishes alpha-synuclein neurotoxicity in a rat model of Parkinson disease. *Mol. Ther.* 20 1327–1337. 10.1038/mt.2012.28 22434142 PMC3392977

[B57] Grassi D. Howard S. Zhou M. Diaz-Perez N. Urban N. T. Guerrero-Given D. (2018). Identification of a highly neurotoxic alpha-synuclein species inducing mitochondrial damage and mitophagy in Parkinson’s disease. *Proc. Natl. Acad. Sci. U.S.A.* 115 E2634–E2643. 10.1073/pnas.1713849115 29487216 PMC5856519

[B58] Gupta S. Biswas J. Gupta P. Singh A. Tiwari S. Mishra A. (2019). Salubrinal attenuates nitric oxide mediated PERK:IRE1alpha: ATF-6 signaling and DNA damage in neuronal cells. *Neurochem. Int.* 131:104581. 10.1016/j.neuint.2019.104581 31639405

[B59] Harding H. P. Novoa I. Zhang Y. Zeng H. Wek R. Schapira M. (2000). Regulated translation initiation controls stress-induced gene expression in mammalian cells. *Mol. Cell* 6 1099–1108. 10.1016/s1097-2765(00)00108-8 11106749

[B60] Haze K. Yoshida H. Yanagi H. Yura T. Mori K. (1999). Mammalian transcription factor ATF6 is synthesized as a transmembrane protein and activated by proteolysis in response to endoplasmic reticulum stress. *Mol. Biol. Cell* 10 3787–3799. 10.1091/mbc.10.11.3787 10564271 PMC25679

[B61] Heman-Ackah S. M. Manzano R. Hoozemans J. J. M. Scheper W. Flynn R. Haerty W. (2017). Alpha-synuclein induces the unfolded protein response in Parkinson’s disease SNCA triplication iPSC-derived neurons. *Hum. Mol. Genet.* 26 4441–4450. 10.1093/hmg/ddx331 28973645 PMC5886237

[B62] Hetz C. Papa F. R. (2018). The unfolded protein response and cell fate control. *Mol. Cell* 69 169–181. 10.1016/j.molcel.2017.06.017 29107536

[B63] Hillary R. F. FitzGerald U. (2018). A lifetime of stress: ATF6 in development and homeostasis. *J. Biomed. Sci.* 25:48. 10.1186/s12929-018-0453-1 29801500 PMC5968583

[B64] Ho D. H. Kim H. Nam D. Sim H. Kim J. Kim H. G. (2018). LRRK2 impairs autophagy by mediating phosphorylation of leucyl-tRNA synthetase. *Cell Biochem. Funct.* 36 431–442. 10.1002/cbf.3364 30411383

[B65] Ho P. W. Leung C. T. Liu H. Pang S. Y. Lam C. S. Xian J. (2020). Age-dependent accumulation of oligomeric SNCA/alpha-synuclein from impaired degradation in mutant LRRK2 knockin mouse model of Parkinson disease: role for therapeutic activation of chaperone-mediated autophagy (CMA). *Autophagy* 16 347–370. 10.1080/15548627.2019.1603545 30983487 PMC6984454

[B66] Holtz W. A. O’Malley K. L. (2003). Parkinsonian mimetics induce aspects of unfolded protein response in death of dopaminergic neurons. *J. Biol. Chem.* 278 19367–19377. 10.1074/jbc.M211821200 12598533

[B67] Hoozemans J. J. van Haastert E. S. Eikelenboom P. de Vos R. A. Rozemuller J. M. Scheper W. (2007). Activation of the unfolded protein response in Parkinson’s disease. *Biochem. Biophys. Res. Commun.* 354 707–711. 10.1016/j.bbrc.2007.01.043 17254549

[B68] Hoozemans J. J. van Haastert E. S. Nijholt D. A. Rozemuller A. J. Scheper W. (2012). Activation of the unfolded protein response is an early event in Alzheimer’s and Parkinson’s disease. *Neurodegener. Dis.* 10 212–215. 10.1159/000334536 22302012

[B69] Hou X. Fiesel F. C. Truban D. Castanedes Casey M. Lin W. L. Soto A. I. (2018). Age- and disease-dependent increase of the mitophagy marker phospho-ubiquitin in normal aging and Lewy body disease. *Autophagy* 14 1404–1418. 10.1080/15548627.2018.1461294 29947276 PMC6372017

[B70] Hou X. Watzlawik J. O. Fiesel F. C. Springer W. (2020). Autophagy in Parkinson’s disease. *J. Mol. Biol.* 432 2651–2672. 10.1016/j.jmb.2020.01.037 32061929 PMC7211126

[B71] Imai Y. Soda M. Inoue H. Hattori N. Mizuno Y. Takahashi R. (2001). An unfolded putative transmembrane polypeptide, which can lead to endoplasmic reticulum stress, is a substrate of Parkin. *Cell* 105 891–902. 10.1016/s0092-8674(01)00407-x 11439185

[B72] Imai Y. Soda M. Takahashi R. (2000). Parkin suppresses unfolded protein stress-induced cell death through its E3 ubiquitin-protein ligase activity. *J. Biol. Chem.* 275 35661–35664. 10.1074/jbc.C000447200 10973942

[B73] Iurlaro R. Munoz-Pinedo C. (2016). Cell death induced by endoplasmic reticulum stress. *FEBS J.* 283 2640–2652. 10.1111/febs.13598 26587781

[B74] Iwata A. Maruyama M. Kanazawa I. Nukina N. (2001). alpha-Synuclein affects the MAPK pathway and accelerates cell death. *J. Biol. Chem.* 276 45320–45329. 10.1074/jbc.M103736200 11560921

[B75] Jiang P. Gan M. Ebrahim A. S. Lin W. L. Melrose H. L. Yen S. H. (2010). ER stress response plays an important role in aggregation of alpha-synuclein. *Mol. Neurodegener.* 5:56. 10.1186/1750-1326-5-56 21144044 PMC3016345

[B76] Jiang T. F. Zhang Y. J. Zhou H. Y. Wang H. M. Tian L. P. Liu J. (2013). Curcumin ameliorates the neurodegenerative pathology in A53T alpha-synuclein cell model of Parkinson’s disease through the downregulation of mTOR/p70S6K signaling and the recovery of macroautophagy. *J. Neuroimmune Pharmacol.* 8 356–369. 10.1007/s11481-012-9431-7 23325107

[B77] Jiao F. J. Wang Q. Z. Zhang P. Yan J. G. Zhang Z. He F. (2017). CDK5-mediated phosphorylation of XBP1s contributes to its nuclear translocation and activation in MPP(+)-induced Parkinson’s disease model. *Sci. Rep.* 7:5622. 10.1038/s41598-017-06012-6 28717189 PMC5514026

[B78] Jovanovic-Tucovic M. Harhaji-Trajkovic L. Dulovic M. Tovilovic-Kovacevic G. Zogovic N. Jeremic M. (2019). AMP-activated protein kinase inhibits MPP+-induced oxidative stress and apoptotic death of SH-SY5Y cells through sequential stimulation of Akt and autophagy. *Eur. J. Pharmacol.* 863:172677. 10.1016/j.ejphar.2019.172677 31542478

[B79] Kalia L. V. Lang A. E. (2015). Parkinson’s disease. *Lancet* 386 896–912. 10.1016/S0140-6736(14)61393-3 25904081

[B80] Kalogeropulou A. F. Zhao J. Bolliger M. F. Memou A. Narasimha S. Molitor T. P. (2018). P62/SQSTM1 is a novel leucine-rich repeat kinase 2 (LRRK2) substrate that enhances neuronal toxicity. *Biochem. J.* 475 1271–1293. 10.1042/BCJ20170699 29519959

[B81] Kalvakolanu D. V. Gade P. (2012). IFNG and autophagy: a critical role for the ER-stress mediator ATF6 in controlling bacterial infections. *Autophagy* 8 1673–1674. 10.4161/auto.21403 22874566 PMC3494595

[B82] Karabiyik C. Lee M. J. Rubinsztein D. C. (2017). Autophagy impairment in Parkinson’s disease. *Essays Biochem.* 61 711–720. 10.1042/EBC20170023 29233880

[B83] Kitada T. Asakawa S. Hattori N. Matsumine H. Yamamura Y. Minoshima S. (1998). Mutations in the parkin gene cause autosomal recessive juvenile parkinsonism. *Nature* 392 605–608. 10.1038/33416 9560156

[B84] Korennykh A. Walter P. (2012). Structural basis of the unfolded protein response. *Annu. Rev. Cell Dev. Biol.* 28 251–277. 10.1146/annurev-cellbio-101011-155826 23057742

[B85] Kouroku Y. Fujita E. Tanida I. Ueno T. Isoai A. Kumagai H. (2007). ER stress (PERK/eIF2alpha phosphorylation) mediates the polyglutamine-induced LC3 conversion, an essential step for autophagy formation. *Cell Death Differ.* 14 230–239. 10.1038/sj.cdd.4401984 16794605

[B86] Lamark T. Kirkin V. Dikic I. Johansen T. (2009). NBR1 and p62 as cargo receptors for selective autophagy of ubiquitinated targets. *Cell Cycle* 8 1986–1990. 10.4161/cc.8.13.8892 19502794

[B87] Ledesma M. D. Galvan C. Hellias B. Dotti C. Jensen P. H. (2002). Astrocytic but not neuronal increased expression and redistribution of parkin during unfolded protein stress. *J. Neurochem.* 83 1431–1440. 10.1046/j.1471-4159.2002.01253.x 12472897

[B88] Lee D. H. Kim D. Kim S. T. Jeong S. Kim J. L. Shim S. M. (2018). PARK7 modulates autophagic proteolysis through binding to the N-terminally arginylated form of the molecular chaperone HSPA5. *Autophagy* 14 1870–1885. 10.1080/15548627.2018.1491212 29976090 PMC6152518

[B89] Lee J. H. Han J. H. Kim H. Park S. M. Joe E. H. Jou I. (2019). Parkinson’s disease-associated LRRK2-G2019S mutant acts through regulation of SERCA activity to control ER stress in astrocytes. *Acta Neuropathol. Commun.* 7:68. 10.1186/s40478-019-0716-4 31046837 PMC6498585

[B90] Lehtonen S. Sonninen T. M. Wojciechowski S. Goldsteins G. Koistinaho J. (2019). Dysfunction of cellular proteostasis in Parkinson’s disease. *Front. Neurosci.* 13:457. 10.3389/fnins.2019.00457 31133790 PMC6524622

[B91] Levine B. Kroemer G. (2019). Biological functions of autophagy genes: a disease perspective. *Cell* 176 11–42. 10.1016/j.cell.2018.09.048 30633901 PMC6347410

[B92] Li D. D. Wang L. L. Deng R. Tang J. Shen Y. Guo J. F. (2009). The pivotal role of c-Jun NH2-terminal kinase-mediated Beclin 1 expression during anticancer agents-induced autophagy in cancer cells. *Oncogene* 28 886–898. 10.1038/onc.2008.441 19060920

[B93] Li L. Hu G. K. (2015). Pink1 protects cortical neurons from thapsigargin-induced oxidative stress and neuronal apoptosis. *Biosci. Rep.* 35:e00174. 10.1042/BSR20140104 25608948 PMC4340272

[B94] Li T. Le W. (2020). Biomarkers for Parkinson’s disease: how good are they? *Neurosci. Bull.* 36 183–194. 10.1007/s12264-019-00433-1 31646434 PMC6977795

[B95] Li W. Zhu J. Dou J. She H. Tao K. Xu H. (2017). Phosphorylation of LAMP2A by p38 MAPK couples ER stress to chaperone-mediated autophagy. *Nat. Commun.* 8:1763. 10.1038/s41467-017-01609-x 29176575 PMC5701254

[B96] Lim J. Kim H. W. Youdim M. B. Rhyu I. J. Choe K. M. Oh Y. J. (2011). Binding preference of p62 towards LC3-ll during dopaminergic neurotoxin-induced impairment of autophagic flux. *Autophagy* 7 51–60. 10.4161/auto.7.1.13909 21045561

[B97] Lim J. Lee Y. Jung S. Youdim M. B. Oh Y. J. (2014). Impaired autophagic flux is critically involved in drug-induced dopaminergic neuronal death. *Parkinsonism Relat. Disord.* 20(Suppl. 1) S162–S166. 10.1016/S1353-8020(13)70039-7 24262172

[B98] Liu J. Liu W. Li R. Yang H. (2019). Mitophagy in Parkinson’s disease: from pathogenesis to treatment. *Cells* 8:712. 10.3390/cells8070712 31336937 PMC6678174

[B99] Liu M. Qin L. Wang L. Tan J. Zhang H. Tang J. (2018). alphasynuclein induces apoptosis of astrocytes by causing dysfunction of the endoplasmic reticulumGolgi compartment. *Mol. Med. Rep.* 18 322–332. 10.3892/mmr.2018.9002 29749529 PMC6059687

[B100] Lucking C. B. Durr A. Bonifati V. Vaughan J. De Michele G. Gasser T. (2000). Association between early-onset Parkinson’s disease and mutations in the parkin gene. *N. Engl. J. Med.* 342 1560–1567. 10.1056/NEJM200005253422103 10824074

[B101] Madureira M. Connor-Robson N. Wade-Martins R. (2020). LRRK2: Autophagy and Lysosomal activity. *Front. Neurosci.* 14:498. 10.3389/fnins.2020.00498 32523507 PMC7262160

[B102] Malkus K. A. Ischiropoulos H. (2012). Regional deficiencies in chaperone-mediated autophagy underlie alpha-synuclein aggregation and neurodegeneration. *Neurobiol. Dis.* 46 732–744. 10.1016/j.nbd.2012.03.017 22426402 PMC3352979

[B103] Manie S. N. Lebeau J. Chevet E. (2014). Cellular mechanisms of endoplasmic reticulum stress signaling in health and disease. 3. Orchestrating the unfolded protein response in oncogenesis: an update. *Am. J. Physiol. Cell Physiol.* 307 C901–C907. 10.1152/ajpcell.00292.2014 25186011

[B104] Margariti A. Li H. Chen T. Martin D. Vizcay-Barrena G. Alam S. (2013). XBP1 mRNA splicing triggers an autophagic response in endothelial cells through BECLIN-1 transcriptional activation. *J. Biol. Chem.* 288 859–872. 10.1074/jbc.M112.412783 23184933 PMC3543035

[B105] Martinez A. Lopez N. Gonzalez C. Hetz C. (2019). Targeting of the unfolded protein response (UPR) as therapy for Parkinson’s disease. *Biol. Cell.* 111 161–168. 10.1111/boc.201800068 30860281

[B106] Martinez-Vicente M. Talloczy Z. Kaushik S. Massey A. C. Mazzulli J. Mosharov E. V. (2008). Dopamine-modified alpha-synuclein blocks chaperone-mediated autophagy. *J. Clin. Invest.* 118 777–788. 10.1172/JCI32806 18172548 PMC2157565

[B107] Martini-Stoica H. Xu Y. Ballabio A. Zheng H. (2016). The Autophagy-Lysosomal pathway in neurodegeneration: a TFEB perspective. *Trends Neurosci.* 39 221–234. 10.1016/j.tins.2016.02.002 26968346 PMC4928589

[B108] Meares G. P. Hughes K. J. Naatz A. Papa F. R. Urano F. Hansen P. A. (2011). IRE1-dependent activation of AMPK in response to nitric oxide. *Mol. Cell. Biol.* 31 4286–4297. 10.1128/MCB.05668-11 21896783 PMC3209336

[B109] Mencke P. Boussaad I. Romano C. D. Kitami T. Linster C. L. Kruger R. (2021). The Role of DJ-1 in cellular metabolism and pathophysiological implications for Parkinson’s disease. *Cells* 10:347. 10.3390/cells10020347 33562311 PMC7915027

[B110] Mercado G. Castillo V. Soto P. Lopez N. Axten J. M. Sardi S. P. (2018). Targeting PERK signaling with the small molecule GSK2606414 prevents neurodegeneration in a model of Parkinson’s disease. *Neurobiol. Dis.* 112 136–148. 10.1016/j.nbd.2018.01.004 29355603

[B111] Miyara M. Kotake Y. Tokunaga W. Sanoh S. Ohta S. (2016). Mild MPP(+) exposure impairs autophagic degradation through a novel lysosomal acidity-independent mechanism. *J. Neurochem.* 139 294–308. 10.1111/jnc.13700 27309572

[B112] Moors T. E. Hoozemans J. J. Ingrassia A. Beccari T. Parnetti L. Chartier-Harlin M. C. (2017). Therapeutic potential of autophagy-enhancing agents in Parkinson’s disease. *Mol. Neurodegener.* 12:11. 10.1186/s13024-017-0154-3 28122627 PMC5267440

[B113] Moors T. E. Paciotti S. Ingrassia A. Quadri M. Breedveld G. Tasegian A. (2019). Characterization of brain Lysosomal activities in GBA-related and sporadic Parkinson’s disease and dementia with Lewy bodies. *Mol. Neurobiol.* 56 1344–1355. 10.1007/s12035-018-1090-0 29948939 PMC6400877

[B114] Murphy K. E. Gysbers A. M. Abbott S. K. Spiro A. S. Furuta A. Cooper A. (2015). Lysosomal-associated membrane protein 2 isoforms are differentially affected in early Parkinson’s disease. *Mov. Disord.* 30 1639–1647. 10.1002/mds.26141 25594542

[B115] Nakamura S. Yoshimori T. (2017). New insights into autophagosome-lysosome fusion. *J. Cell Sci.* 130 1209–1216. 10.1242/jcs.196352 28302910

[B116] Nascimbeni A. C. Codogno P. Morel E. (2017). Phosphatidylinositol-3-phosphate in the regulation of autophagy membrane dynamics. *FEBS J.* 284 1267–1278. 10.1111/febs.13987 27973739

[B117] Nash Y. Schmukler E. Trudler D. Pinkas-Kramarski R. Frenkel D. (2017). DJ-1 deficiency impairs autophagy and reduces alpha-synuclein phagocytosis by microglia. *J. Neurochem.* 143 584–594. 10.1111/jnc.14222 28921554

[B118] Nguyen T. N. Padman B. S. Lazarou M. (2016). Deciphering the molecular signals of PINK1/Parkin mitophagy. *Trends Cell Biol.* 26 733–744. 10.1016/j.tcb.2016.05.008 27291334

[B119] Ning B. Zhang Q. Deng M. Wang N. Fang Y. (2019a). Endoplasmic reticulum stress induced autophagy in 6-OHDA-induced Parkinsonian rats. *Brain Res. Bull.* 146 224–227. 10.1016/j.brainresbull.2019.01.001 30625371

[B120] Ning B. Zhang Q. Wang N. Deng M. Fang Y. (2019b). beta-Asarone regulates er stress and autophagy via inhibition of the PERK/CHOP/Bcl-2/Beclin-1 pathway in 6-OHDA-induced parkinsonian rats. *Neurochem. Res.* 44 1159–1166. 10.1007/s11064-019-02757-w 30796752

[B121] Noda N. N. Inagaki F. (2015). Mechanisms of autophagy. *Annu. Rev. Biophys.* 44 101–122. 10.1146/annurev-biophys-060414-034248 25747593

[B122] Novoa I. Zeng H. Harding H. P. Ron D. (2001). Feedback inhibition of the unfolded protein response by GADD34-mediated dephosphorylation of eIF2alpha. *J. Cell Biol.* 153 1011–1022. 10.1083/jcb.153.5.1011 11381086 PMC2174339

[B123] Oakes S. A. Papa F. R. (2015). The role of endoplasmic reticulum stress in human pathology. *Annu. Rev. Pathol.* 10 173–194. 10.1146/annurev-pathol-012513-104649 25387057 PMC5568783

[B124] Ogata M. Hino S. Saito A. Morikawa K. Kondo S. Kanemoto S. (2006). Autophagy is activated for cell survival after endoplasmic reticulum stress. *Mol. Cell. Biol.* 26 9220–9231. 10.1128/MCB.01453-06 17030611 PMC1698520

[B125] Orenstein S. J. Kuo S. H. Tasset I. Arias E. Koga H. Fernandez-Carasa I. (2013). Interplay of LRRK2 with chaperone-mediated autophagy. *Nat. Neurosci.* 16 394–406. 10.1038/nn.3350 23455607 PMC3609872

[B126] Paiva I. Jain G. Lazaro D. F. Jercic K. G. Hentrich T. Kerimoglu C. (2018). Alpha-synuclein deregulates the expression of COL4A2 and impairs ER-Golgi function. *Neurobiol. Dis.* 119 121–135. 10.1016/j.nbd.2018.08.001 30092270

[B127] Pandey V. K. Mathur A. Kakkar P. (2019). Emerging role of Unfolded Protein Response (UPR) mediated proteotoxic apoptosis in diabetes. *Life Sci.* 216 246–258. 10.1016/j.lfs.2018.11.041 30471281

[B128] Park S. Han S. Choi I. Kim B. Park S. P. Joe E. H. (2016). Interplay between Leucine-rich repeat Kinase 2 (LRRK2) and p62/SQSTM-1 in selective Autophagy. *PLoS One* 11:e0163029. 10.1371/journal.pone.0163029 27631370 PMC5025236

[B129] Pattingre S. Tassa A. Qu X. Garuti R. Liang X. H. Mizushima N. (2005). Bcl-2 antiapoptotic proteins inhibit Beclin 1-dependent autophagy. *Cell* 122 927–939. 10.1016/j.cell.2005.07.002 16179260

[B130] Poewe W. Seppi K. Tanner C. M. Halliday G. M. Brundin P. Volkmann J. (2017). Parkinson disease. *Nat. Rev. Dis. Primers* 3:17013. 10.1038/nrdp.2017.13 28332488

[B131] Puschmann A. (2013). Monogenic Parkinson’s disease and parkinsonism: clinical phenotypes and frequencies of known mutations. *Parkinsonism Relat. Disord.* 19 407–415. 10.1016/j.parkreldis.2013.01.020 23462481

[B132] Ramalingam M. Huh Y. J. Lee Y. I. (2019). The impairments of alpha-Synuclein and mechanistic target of rapamycin in rotenone-induced SH-SY5Y cells and mice model of Parkinson’s disease. *Front. Neurosci.* 13:1028. 10.3389/fnins.2019.01028 31611767 PMC6769080

[B133] Rashid H. O. Yadav R. K. Kim H. R. Chae H. J. (2015). ER stress: autophagy induction, inhibition and selection. *Autophagy* 11 1956–1977. 10.1080/15548627.2015.1091141 26389781 PMC4824587

[B134] Ren H. Fu K. Mu C. Li B. Wang D. Wang G. (2010). DJ-1, a cancer and Parkinson’s disease associated protein, regulates autophagy through JNK pathway in cancer cells. *Cancer Lett.* 297 101–108. 10.1016/j.canlet.2010.05.001 20510502

[B135] Rouschop K. M. van den Beucken T. Dubois L. Niessen H. Bussink J. Savelkouls K. (2010). The unfolded protein response protects human tumor cells during hypoxia through regulation of the autophagy genes MAP1LC3B and ATG5. *J. Clin. Invest.* 120 127–141. 10.1172/JCI40027 20038797 PMC2798689

[B136] Rubinsztein D. C. Nixon R. A. (2010). Rapamycin induces autophagic flux in neurons. *Proc. Natl. Acad. Sci. U.S.A.* 107:E181. 10.1073/pnas.1014633107 21115811 PMC3000262

[B137] Russell R. C. Tian Y. Yuan H. Park H. W. Chang Y. Y. Kim J. (2013). ULK1 induces autophagy by phosphorylating Beclin-1 and activating VPS34 lipid kinase. *Nat. Cell Biol.* 15 741–750. 10.1038/ncb2757 23685627 PMC3885611

[B138] Ryu E. J. Harding H. P. Angelastro J. M. Vitolo O. V. Ron D. Greene L. A. (2002). Endoplasmic reticulum stress and the unfolded protein response in cellular models of Parkinson’s disease. *J. Neurosci.* 22 10690–10698. 12486162 10.1523/JNEUROSCI.22-24-10690.2002 PMC6758450

[B139] Rzymski T. Milani M. Pike L. Buffa F. Mellor H. R. Winchester L. (2010). Regulation of autophagy by ATF4 in response to severe hypoxia. *Oncogene* 29 4424–4435. 10.1038/onc.2010.191 20514020

[B140] Sado M. Yamasaki Y. Iwanaga T. Onaka Y. Ibuki T. Nishihara S. (2009). Protective effect against Parkinson’s disease-related insults through the activation of XBP1. *Brain Res.* 1257 16–24. 10.1016/j.brainres.2008.11.104 19135031

[B141] Salazar M. Carracedo A. Salanueva I. J. Hernandez-Tiedra S. Lorente M. Egia A. (2009). Cannabinoid action induces autophagy-mediated cell death through stimulation of ER stress in human glioma cells. *J. Clin. Invest.* 119 1359–1372. 10.1172/jci37948 19425170 PMC2673842

[B142] Selvaraj S. Sun Y. Watt J. A. Wang S. Lei S. Birnbaumer L. (2012). Neurotoxin-induced ER stress in mouse dopaminergic neurons involves downregulation of TRPC1 and inhibition of AKT/mTOR signaling. *J. Clin. Invest.* 122 1354–1367. 10.1172/JCI61332 22446186 PMC3314472

[B143] Senft D. Ronai Z. A. (2015). UPR, autophagy, and mitochondria crosstalk underlies the ER stress response. *Trends Biochem. Sci.* 40 141–148. 10.1016/j.tibs.2015.01.002 25656104 PMC4340752

[B144] Shahmoradian S. H. Lewis A. J. Genoud C. Hench J. Moors T. E. Navarro P. P. (2019). Lewy pathology in Parkinson’s disease consists of crowded organelles and lipid membranes. *Nat. Neurosci.* 22 1099–1109. 10.1038/s41593-019-0423-2 31235907

[B145] Shen J. Chen X. Hendershot L. Prywes R. (2002). ER stress regulation of ATF6 localization by dissociation of BiP/GRP78 binding and unmasking of Golgi localization signals. *Dev. Cell* 3 99–111. 10.1016/s1534-5807(02)00203-4 12110171

[B146] Shimizu S. (2018). Biological roles of alternative Autophagy. *Mol. Cells* 41 50–54. 10.14348/molcells.2018.2215 29370693 PMC5792713

[B147] Shimura H. Hattori N. Kubo S. Mizuno Y. Asakawa S. Minoshima S. (2000). Familial Parkinson disease gene product, parkin, is a ubiquitin-protein ligase. *Nat. Genet.* 25 302–305. 10.1038/77060 10888878

[B148] Shulman J. M. De Jager P. L. Feany M. B. (2011). Parkinson’s disease: genetics and pathogenesis. *Annu. Rev. Pathol.* 6 193–222. 10.1146/annurev-pathol-011110-130242 21034221

[B149] Simola N. Morelli M. Carta A. R. (2007). The 6-hydroxydopamine model of Parkinson’s disease. *Neurotox. Res.* 11 151–167. 10.1007/BF03033565 17449457

[B150] Singh K. Han K. Tilve S. Wu K. Geller H. M. Sack M. N. (2018). Parkin targets NOD2 to regulate astrocyte endoplasmic reticulum stress and inflammation. *Glia* 66 2427–2437. 10.1002/glia.23482 30378174 PMC6275110

[B151] Slodzinski H. Moran L. B. Michael G. J. Wang B. Novoselov S. Cheetham M. E. (2009). Homocysteine-induced endoplasmic reticulum protein (herp) is up-regulated in parkinsonian substantia nigra and present in the core of Lewy bodies. *Clin. Neuropathol.* 28 333–343. 19788048

[B152] Smith W. W. Jiang H. Pei Z. Tanaka Y. Morita H. Sawa A. (2005). Endoplasmic reticulum stress and mitochondrial cell death pathways mediate A53T mutant alpha-synuclein-induced toxicity. *Hum. Mol. Genet.* 14 3801–3811. 10.1093/hmg/ddi396 16239241

[B153] Song J. X. Lu J. H. Liu L. F. Chen L. L. Durairajan S. S. Yue Z. (2014). HMGB1 is involved in autophagy inhibition caused by SNCA/alpha-synuclein overexpression: a process modulated by the natural autophagy inducer corynoxine B. *Autophagy* 10 144–154. 10.4161/auto.26751 24178442 PMC4389868

[B154] Stefanis L. Larsen K. E. Rideout H. J. Sulzer D. Greene L. A. (2001). Expression of A53T mutant but not wild-type alpha-synuclein in PC12 cells induces alterations of the ubiquitin-dependent degradation system, loss of dopamine release, and autophagic cell death. *J. Neurosci.* 21 9549–9560. 11739566 10.1523/JNEUROSCI.21-24-09549.2001 PMC6763041

[B155] Stolz A. Ernst A. Dikic I. (2014). Cargo recognition and trafficking in selective autophagy. *Nat. Cell Biol.* 16 495–501. 10.1038/ncb2979 24875736

[B156] Sugeno N. Takeda A. Hasegawa T. Kobayashi M. Kikuchi A. Mori F. (2008). Serine 129 phosphorylation of alpha-synuclein induces unfolded protein response-mediated cell death. *J. Biol. Chem.* 283 23179–23188. 10.1074/jbc.M802223200 18562315

[B157] Swerdlow N. S. Wilkins H. M. (2020). Mitophagy and the brain. *Int. J. Mol. Sci.* 21:9661. 10.3390/ijms21249661 33352896 PMC7765816

[B158] Takahashi Y. He H. Tang Z. Hattori T. Liu Y. Young M. M. (2018). An autophagy assay reveals the ESCRT-III component CHMP2A as a regulator of phagophore closure. *Nat. Commun.* 9:2855. 10.1038/s41467-018-05254-w 30030437 PMC6054611

[B159] Takahashi Y. Liang X. Hattori T. Tang Z. He H. Chen H. (2019). VPS37A directs ESCRT recruitment for phagophore closure. *J. Cell Biol.* 218 3336–3354. 10.1083/jcb.201902170 31519728 PMC6781443

[B160] Tanji K. Mori F. Kakita A. Takahashi H. Wakabayashi K. (2011). Alteration of autophagosomal proteins (LC3, GABARAP and GATE-16) in Lewy body disease. *Neurobiol. Dis.* 43 690–697. 10.1016/j.nbd.2011.05.022 21684337

[B161] Tekirdag K. Cuervo A. M. (2018). Chaperone-mediated autophagy and endosomal microautophagy: joint by a chaperone. *J. Biol. Chem.* 293 5414–5424. 10.1074/jbc.R117.818237 29247007 PMC5900761

[B162] Tolosa E. Vila M. Klein C. Rascol O. (2020). LRRK2 in Parkinson disease: challenges of clinical trials. *Nat. Rev. Neurol.* 16 97–107. 10.1038/s41582-019-0301-2 31980808

[B163] Tong Q. Wu L. Gao Q. Ou Z. Zhu D. Zhang Y. (2016a). PPARbeta/delta agonist provides neuroprotection by suppression of IRE1alpha-caspase-12-mediated endoplasmic reticulum stress pathway in the rotenone rat model of Parkinson’s disease. *Mol. Neurobiol.* 53 3822–3831. 10.1007/s12035-015-9309-9 26160761

[B164] Tong Q. Wu L. Jiang T. Ou Z. Zhang Y. Zhu D. (2016b). Inhibition of endoplasmic reticulum stress-activated IRE1alpha-TRAF2-caspase-12 apoptotic pathway is involved in the neuroprotective effects of telmisartan in the rotenone rat model of Parkinson’s disease. *Eur. J. Pharmacol.* 776 106–115. 10.1016/j.ejphar.2016.02.042 26879867

[B165] Torres-Odio S. Key J. Hoepken H. H. Canet-Pons J. Valek L. Roller B. (2017). Progression of pathology in PINK1-deficient mouse brain from splicing via ubiquitination, ER stress, and mitophagy changes to neuroinflammation. *J. Neuroinflamm.* 14:154. 10.1186/s12974-017-0928-0 28768533 PMC5541666

[B166] Toyofuku T. Okamoto Y. Ishikawa T. Sasawatari S. Kumanogoh A. (2020). LRRK2 regulates endoplasmic reticulum-mitochondrial tethering through the PERK-mediated ubiquitination pathway. *EMBO J.* 39:e100875. 10.15252/embj.2018100875 31821596 PMC6960452

[B167] Tsuboyama K. Koyama-Honda I. Sakamaki Y. Koike M. Morishita H. Mizushima N. (2016). The ATG conjugation systems are important for degradation of the inner autophagosomal membrane. *Science* 354 1036–1041. 10.1126/science.aaf6136 27885029

[B168] Turano C. Coppari S. Altieri F. Ferraro A. (2002). Proteins of the PDI family: unpredicted non-ER locations and functions. *J. Cell. Physiol.* 193 154–163. 10.1002/jcp.10172 12384992

[B169] Urano F. Wang X. Bertolotti A. Zhang Y. Chung P. Harding H. P. (2000). Coupling of stress in the ER to activation of JNK protein kinases by transmembrane protein kinase IRE1. *Science* 287 664–666. 10.1126/science.287.5453.664 10650002

[B170] Valdes P. Mercado G. Vidal R. L. Molina C. Parsons G. Court F. A. (2014). Control of dopaminergic neuron survival by the unfolded protein response transcription factor XBP1. *Proc. Natl. Acad. Sci. U.S.A.* 111 6804–6809. 10.1073/pnas.1321845111 24753614 PMC4020088

[B171] Valente E. M. Abou-Sleiman P. M. Caputo V. Muqit M. M. Harvey K. Gispert S. (2004a). Hereditary early-onset Parkinson’s disease caused by mutations in PINK1. *Science* 304 1158–1160. 10.1126/science.1096284 15087508

[B172] Valente E. M. Salvi S. Ialongo T. Marongiu R. Elia A. E. Caputo V. (2004b). PINK1 mutations are associated with sporadic early-onset parkinsonism. *Ann. Neurol.* 56 336–341. 10.1002/ana.20256 15349860

[B173] Verfaillie T. Salazar M. Velasco G. Agostinis P. (2010). Linking ER stress to autophagy: potential implications for cancer therapy. *Int. J. Cell Biol.* 2010:930509. 10.1155/2010/930509 20145727 PMC2817393

[B174] Vidal R. L. Figueroa A. Court F. A. Thielen P. Molina C. Wirth C. (2012). Targeting the UPR transcription factor XBP1 protects against Huntington’s disease through the regulation of FoxO1 and autophagy. *Hum. Mol. Genet.* 21 2245–2262. 10.1093/hmg/dds040 22337954 PMC3335312

[B175] Vitte J. Traver S. Maues De Paula A. Lesage S. Rovelli G. Corti O. (2010). Leucine-rich repeat kinase 2 is associated with the endoplasmic reticulum in dopaminergic neurons and accumulates in the core of Lewy bodies in Parkinson disease. *J. Neuropathol. Exp. Neurol.* 69 959–972. 10.1097/NEN.0b013e3181efc01c 20720502

[B176] Walter P. Ron D. (2011). The unfolded protein response: from stress pathway to homeostatic regulation. *Science* 334 1081–1086. 10.1126/science.1209038 22116877

[B177] Wang M. Kaufman R. J. (2016). Protein misfolding in the endoplasmic reticulum as a conduit to human disease. *Nature* 529 326–335. 10.1038/nature17041 26791723

[B178] Wang R. Sun H. Ren H. Wang G. (2020a). alpha-Synuclein aggregation and transmission in Parkinson’s disease: a link to mitochondria and lysosome. *Sci. China Life Sci.* 63 1850–1859. 10.1007/s11427-020-1756-9 32681494

[B179] Wang R. Sun H. Wang G. Ren H. (2020b). Imbalance of Lysine Acetylation contributes to the pathogenesis of Parkinson’s disease. *Int. J. Mol. Sci.* 21:7182. 10.3390/ijms21197182 33003340 PMC7582258

[B180] Wang X. Zhai H. Wang F. (2018). 6-OHDA induces oxidation of F-box protein Fbw7beta by chaperone-mediated Autophagy in Parkinson’s model. *Mol. Neurobiol.* 55 4825–4833. 10.1007/s12035-017-0686-0 28733899

[B181] Wei Y. Pattingre S. Sinha S. Bassik M. Levine B. (2008). JNK1-mediated phosphorylation of Bcl-2 regulates starvation-induced autophagy. *Mol. Cell* 30 678–688. 10.1016/j.molcel.2008.06.001 18570871 PMC2478643

[B182] Weidberg H. Shvets E. Shpilka T. Shimron F. Shinder V. Elazar Z. (2010). LC3 and GATE-16/GABARAP subfamilies are both essential yet act differently in autophagosome biogenesis. *EMBO J.* 29 1792–1802. 10.1038/emboj.2010.74 20418806 PMC2885923

[B183] Winslow A. R. Chen C. W. Corrochano S. Acevedo-Arozena A. Gordon D. E. Peden A. A. (2010). alpha-Synuclein impairs macroautophagy: implications for Parkinson’s disease. *J. Cell Biol.* 190 1023–1037. 10.1083/jcb.201003122 20855506 PMC3101586

[B184] Wu F. Xu H. D. Guan J. J. Hou Y. S. Gu J. H. Zhen X. C. (2015). Rotenone impairs autophagic flux and lysosomal functions in Parkinson’s disease. *Neuroscience* 284 900–911. 10.1016/j.neuroscience.2014.11.004 25446361

[B185] Wu L. Luo N. Zhao H. R. Gao Q. Lu J. Pan Y. (2014). Salubrinal protects against rotenone-induced SH-SY5Y cell death via ATF4-parkin pathway. *Brain Res.* 1549 52–62. 10.1016/j.brainres.2014.01.003 24418467

[B186] Xie L. Tiong C. X. Bian J. S. (2012). Hydrogen sulfide protects SH-SY5Y cells against 6-hydroxydopamine-induced endoplasmic reticulum stress. *Am. J. Physiol. Cell Physiol.* 303 C81–C91. 10.1152/ajpcell.00281.2011 22555844

[B187] Xilouri M. Vogiatzi T. Vekrellis K. Park D. Stefanis L. (2009). Abberant alpha-synuclein confers toxicity to neurons in part through inhibition of chaperone-mediated autophagy. *PLoS One* 4:e5515. 10.1371/journal.pone.0005515 19436756 PMC2677735

[B188] Xiong N. Xiong J. Jia M. Liu L. Zhang X. Chen Z. (2013). The role of autophagy in Parkinson’s disease: rotenone-based modeling. *Behav. Brain Funct.* 9:13. 10.1186/1744-9081-9-13 23497442 PMC3606411

[B189] Xu C. Y. Kang W. Y. Chen Y. M. Jiang T. F. Zhang J. Zhang L. N. (2017). DJ-1 inhibits alpha-synuclein aggregation by regulating chaperone-mediated Autophagy. *Front. Aging Neurosci.* 9:308. 10.3389/fnagi.2017.00308 29021755 PMC5623690

[B190] Xu J. Zhou Q. Xu W. Cai L. (2012). Endoplasmic reticulum stress and diabetic cardiomyopathy. *Exp. Diabetes Res.* 2012:827971. 10.1155/2012/827971 22144992 PMC3226330

[B191] Yamamoto K. Sato T. Matsui T. Sato M. Okada T. Yoshida H. (2007). Transcriptional induction of mammalian ER quality control proteins is mediated by single or combined action of ATF6alpha and XBP1. *Dev. Cell* 13 365–376. 10.1016/j.devcel.2007.07.018 17765680

[B192] Yamamuro A. Yoshioka Y. Ogita K. Maeda S. (2006). Involvement of endoplasmic reticulum stress on the cell death induced by 6-hydroxydopamine in human neuroblastoma SH-SY5Y cells. *Neurochem. Res.* 31 657–664. 10.1007/s11064-006-9062-6 16770736

[B193] Yang J. Kim K. S. Iyirhiaro G. O. Marcogliese P. C. Callaghan S. M. Qu D. (2019). DJ-1 modulates the unfolded protein response and cell death via upregulation of ATF4 following ER stress. *Cell Death Dis.* 10:135. 10.1038/s41419-019-1354-2 30755590 PMC6372623

[B194] Yokota T. Sugawara K. Ito K. Takahashi R. Ariga H. Mizusawa H. (2003). Down regulation of DJ-1 enhances cell death by oxidative stress, ER stress, and proteasome inhibition. *Biochem. Biophys. Res. Commun.* 312 1342–1348. 10.1016/j.bbrc.2003.11.056 14652021

[B195] Yorimitsu T. Nair U. Yang Z. Klionsky D. J. (2006). Endoplasmic reticulum stress triggers autophagy. *J. Biol. Chem.* 281 30299–30304. 10.1074/jbc.M607007200 16901900 PMC1828866

[B196] Young M. M. Wang H. G. (2018). Sphingolipids as regulators of Autophagy and Endocytic trafficking. *Adv. Cancer Res.* 140 27–60. 10.1016/bs.acr.2018.04.008 30060813

[B197] Yu W. H. Dorado B. Figueroa H. Y. Wang L. Planel E. Cookson M. R. (2009). Metabolic activity determines efficacy of macroautophagic clearance of pathological oligomeric alpha-synuclein. *Am. J. Pathol.* 175 736–747. 10.2353/ajpath.2009.080928 19628769 PMC2716969

[B198] Yuan Y. Cao P. Smith M. A. Kramp K. Huang Y. Hisamoto N. (2011). Dysregulated LRRK2 signaling in response to endoplasmic reticulum stress leads to dopaminergic neuron degeneration in *C. elegans*. *PLoS One* 6:e22354. 10.1371/journal.pone.0022354 21857923 PMC3153934

[B199] Yung H. W. Charnock-Jones D. S. Burton G. J. (2011). Regulation of AKT phosphorylation at Ser473 and Thr308 by endoplasmic reticulum stress modulates substrate specificity in a severity dependent manner. *PLoS One* 6:e17894. 10.1371/journal.pone.0017894 21445305 PMC3061875

[B200] Zalckvar E. Berissi H. Mizrachy L. Idelchuk Y. Koren I. Eisenstein M. (2009). DAP-kinase-mediated phosphorylation on the BH3 domain of beclin 1 promotes dissociation of beclin 1 from Bcl-XL and induction of autophagy. *EMBO Rep.* 10 285–292. 10.1038/embor.2008.246 19180116 PMC2658558

[B201] Zhang X. Yuan Y. Jiang L. Zhang J. Gao J. Shen Z. (2014). Endoplasmic reticulum stress induced by tunicamycin and thapsigargin protects against transient ischemic brain injury: involvement of PARK2-dependent mitophagy. *Autophagy* 10 1801–1813. 10.4161/auto.32136 25126734 PMC4198364

[B202] Zhao Y. Li X. Cai M. Y. Ma K. Yang J. Zhou J. (2013). XBP-1u suppresses autophagy by promoting the degradation of FoxO1 in cancer cells. *Cell Res.* 23 491–507. 10.1038/cr.2013.2 23277279 PMC3616429

[B203] Zhao Y. G. Chen Y. Miao G. Zhao H. Qu W. Li D. (2017). The ER-Localized transmembrane protein EPG-3/VMP1 regulates SERCA activity to control ER-isolation membrane contacts for Autophagosome formation. *Mol. Cell* 67 974–989.e976. 10.1016/j.molcel.2017.08.005 28890335

[B204] Zhao Y. G. Zhang H. (2019). Autophagosome maturation: an epic journey from the ER to lysosomes. *J. Cell Biol.* 218 757–770. 10.1083/jcb.201810099 30578282 PMC6400552

[B205] Zhou Y. Lee J. Reno C. M. Sun C. Park S. W. Chung J. (2011). Regulation of glucose homeostasis through a XBP-1-FoxO1 interaction. *Nat. Med.* 17 356–365. 10.1038/nm.2293 21317886 PMC3897616

[B206] Zhu J. H. Guo F. Shelburne J. Watkins S. Chu C. T. (2003). Localization of phosphorylated ERK/MAP kinases to mitochondria and autophagosomes in Lewy body diseases. *Brain Pathol.* 13 473–481. 10.1111/j.1750-3639.2003.tb00478.x 14655753 PMC1911206

[B207] Zhu J. H. Gusdon A. M. Cimen H. Van Houten B. Koc E. Chu C. T. (2012). Impaired mitochondrial biogenesis contributes to depletion of functional mitochondria in chronic MPP+ toxicity: dual roles for ERK1/2. *Cell Death Dis.* 3:e312. 10.1038/cddis.2012.46 22622131 PMC3366080

[B208] Zhu J. H. Horbinski C. Guo F. Watkins S. Uchiyama Y. Chu C. T. (2007). Regulation of autophagy by extracellular signal-regulated protein kinases during 1-methyl-4-phenylpyridinium-induced cell death. *Am. J. Pathol.* 170 75–86. 10.2353/ajpath.2007.060524 17200184 PMC1762689

[B209] Zhu X. Huang L. Gong J. Shi C. Wang Z. Ye B. (2017). NF-kappaB pathway link with ER stress-induced autophagy and apoptosis in cervical tumor cells. *Cell Death Discov.* 3:17059. 10.1038/cddiscovery.2017.59 28904818 PMC5592653

[B210] Zhu Y. Wang C. Yu M. Cui J. Liu L. Xu Z. (2013). ULK1 and JNK are involved in mitophagy incurred by LRRK2 G2019S expression. *Protein Cell* 4 711–721. 10.1007/s13238-013-3910-3 27023913 PMC4875534

